# Structure and function of the Ts2631 endolysin of *Thermus scotoductus* phage vB_Tsc2631 with unique N-terminal extension used for peptidoglycan binding

**DOI:** 10.1038/s41598-018-37417-6

**Published:** 2019-02-04

**Authors:** Magdalena Plotka, Enea Sancho-Vaello, Sebastian Dorawa, Anna-Karina Kaczorowska, Lukasz P. Kozlowski, Tadeusz Kaczorowski, Kornelius Zeth

**Affiliations:** 1 Laboratory of Extremophiles Biology, Department of Microbiology, Faculty of Biology, University of Gdansk, Gdansk, Poland; 2 Unidad de Biofisica, Centro Mixto Consejo Superior de Investigaciones Científicas-Universidad del País Vasco/Euskal Herriko Unibertsitatea (CSIC,UPV/EHU), Leioa, Bizkaia Spain; 3 Collection of Plasmids and Microorganisms, Faculty of Biology, University of Gdansk, Gdansk, Poland; 4 Institute of Informatics, Faculty of Mathematics, Informatics and Mechanics, University of Warsaw, Warsaw, Poland; 5 Department of Science and Environment, Roskilde University, Roskilde, Denmark

## Abstract

To escape from hosts after completing their life cycle, bacteriophages often use endolysins, which degrade bacterial peptidoglycan. While mesophilic phages have been extensively studied, their thermophilic counterparts are not well characterized. Here, we present a detailed analysis of the structure and function of Ts2631 endolysin from thermophilic phage vB_Tsc2631, which is a zinc-dependent amidase. The active site of Ts2631 consists of His30, Tyr58, His131 and Cys139, which are involved in Zn^2+^ coordination and catalysis. We found that the active site residues are necessary for lysis yet not crucial for peptidoglycan binding. To elucidate residues involved in the enzyme interaction with peptidoglycan, we tested single-residue substitution variants and identified Tyr60 and Lys70 as essential residues. Moreover, substitution of Cys80, abrogating disulfide bridge formation, inactivates Ts2631, as do substitutions of His31, Thr32 and Asn85 residues. The endolysin contains a positively charged N-terminal extension of 20 residues that can protrude from the remainder of the enzyme and is crucial for peptidoglycan binding. We show that the deletion of 20 residues from the N-terminus abolished the bacteriolytic activity of the enzyme. Because Ts2631 exhibits intrinsic antibacterial activity and unusual thermal stability, it is perfectly suited as a scaffold for the development of antimicrobial agents.

## Introduction

Upon completing replication and the assembly of new infectious virions, bacteriophages, viruses that infect prokaryotic cells, often rely on their lytic enzymes (endolysins) to release phage progeny from the host. In the cases of most double-stranded DNA bacteriophages targeting Gram-negative bacteria, the escape pathway follows three consecutive steps. Insertion of pore-forming small phage proteins called holins into the bacterial inner membrane first results in its permeabilization and then allows phage lytic enzymes (endolysins) to enter the periplasm^1^. Finally, endolysins degrade the exposed peptidoglycan (PGN) layer before additional phage proteins (spanins) disrupt the outer membrane and enable the descendant virions to be liberated from the lysed bacterial cells^2^. The ability of endolysins to attack peptidoglycan, the most characteristic structure vital for bacterial survival, makes these enzymes an interesting subject to study from a practical point of view, especially in the context of increasing antibiotic resistance. Endolysins also can kill susceptible bacteria when applied exogenously as recombinant proteins^2^. The features of endolysins desired for their function as potential novel antimicrobial agents are their high stability and resistance to proteolysis and chemical denaturation^3,4^. As these physical properties are common among enzymes of thermophilic microorganisms, we focused our research on lytic enzymes encoded by bacteriophages thriving in extreme thermophilic environments.

Bacteriophage vB_Tsc2631 was isolated from a hot spring of the Hveragerði geothermal area, Iceland^5^. This lytic phage can use the thermophilic bacteria *Thermus scotoductus* MAT2631 and *Thermus thermophilus* HB8 as host cells for propagation. The vB_Tsc2631 genome encodes the Ts2631 endolysin (156 aa), an N-acetylmuramoyl-L-alanine type 2 putative amidase that cleaves the amide bond between the sugar moiety and the peptide in PGN structures^5^. The protein is unusually stable, with a melting temperature 99.8 °C that ranks it among the most thermostable enzymes^5,6^.

A comparative *in silico* analysis of the amino acid sequence of Ts2631 endolysin indicated that this enzyme is a structural homolog of phage T7 lysozyme and belongs to a large superfamily including three families of proteins capable of binding bacterial PGN. The first such family is exemplified by the T7 lysozyme itself, the best studied example of type 2 amidases. T7 lysozyme is a globular and monomeric protein possessing a single enzymatically active domain with a Zn^2+^-binding site formed by essential histidines and a cysteine residue in the catalytic center^7^. The second family contains enzymes of bacterial origin acting as autolysins, specifically AmpD-type amidases, which degrade PGN during cell-wall recycling^8^. The third group, called the PGN recognition proteins (PGRPs), was more recently discovered in eukaryotes^9^. They are involved in innate immunity and control the levels of symbiotic microorganisms, but only a few have amidase activity. For example, mammals have four PGRPs, but only one of them (PGLYRP2) is an amidase^10,11^. Nevertheless, all these proteins contain at least one so-called PGRP domain, which is structurally similar to T7 lysozyme and Ts2631 endolysin. Crystal structures of T7 lysozyme and a few PGRPs from *Drosophila melanogaster* and *Homo sapiens* have been determined^10,12–14^.

Here, we report the first crystal structure of an endolysin from a thermophilic bacteriophage. We analyzed the structure and sequence of Ts2631 endolysin and prepared 24 point mutation variants and one N-terminal deletion variant to determine residues that (*i*) are essential for lytic activity and (*ii*) are responsible for PGN binding. In substrate binding analysis, special attention was paid to those residues whose counterparts in PGRPs form the PGN-binding pocket.

The Ts2631 endolysin contains a 20 residue extension at the N-terminus with a unique motif rich in arginines that is not homologous to any other protein sequence present in the UniProt database. The role of the N-terminal region in both the antibacterial and substrate binding activity of Ts2631 endolysin has been investigated. Using this unique N-terminal sequence for the design of fusion proteins might become a platform for the development of novel protein antibiotics targeting Gram-negative bacteria.

## Results

### The structure of Ts2631 endolysin

**Figure 1:**
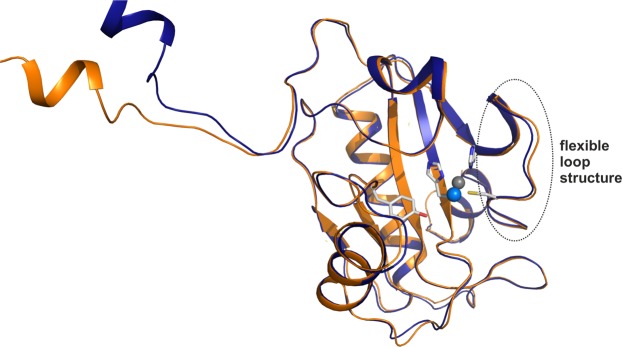
Superposition of the two Ts2631 endolysin monomers in the asymmetric unit. The two independent molecules of Ts2631 endolysin observed in the asymmetric unit superimpose with an r.m.s.d. of 0.6 Å (for residues 16–156; monomers are marked in orange and dark blue). The N-termini of the molecules do not superimpose due to a deviation from perfect non-crystallographic symmetry. The flexible loop structure is indicated by a dotted oval.

**Figure 2:**
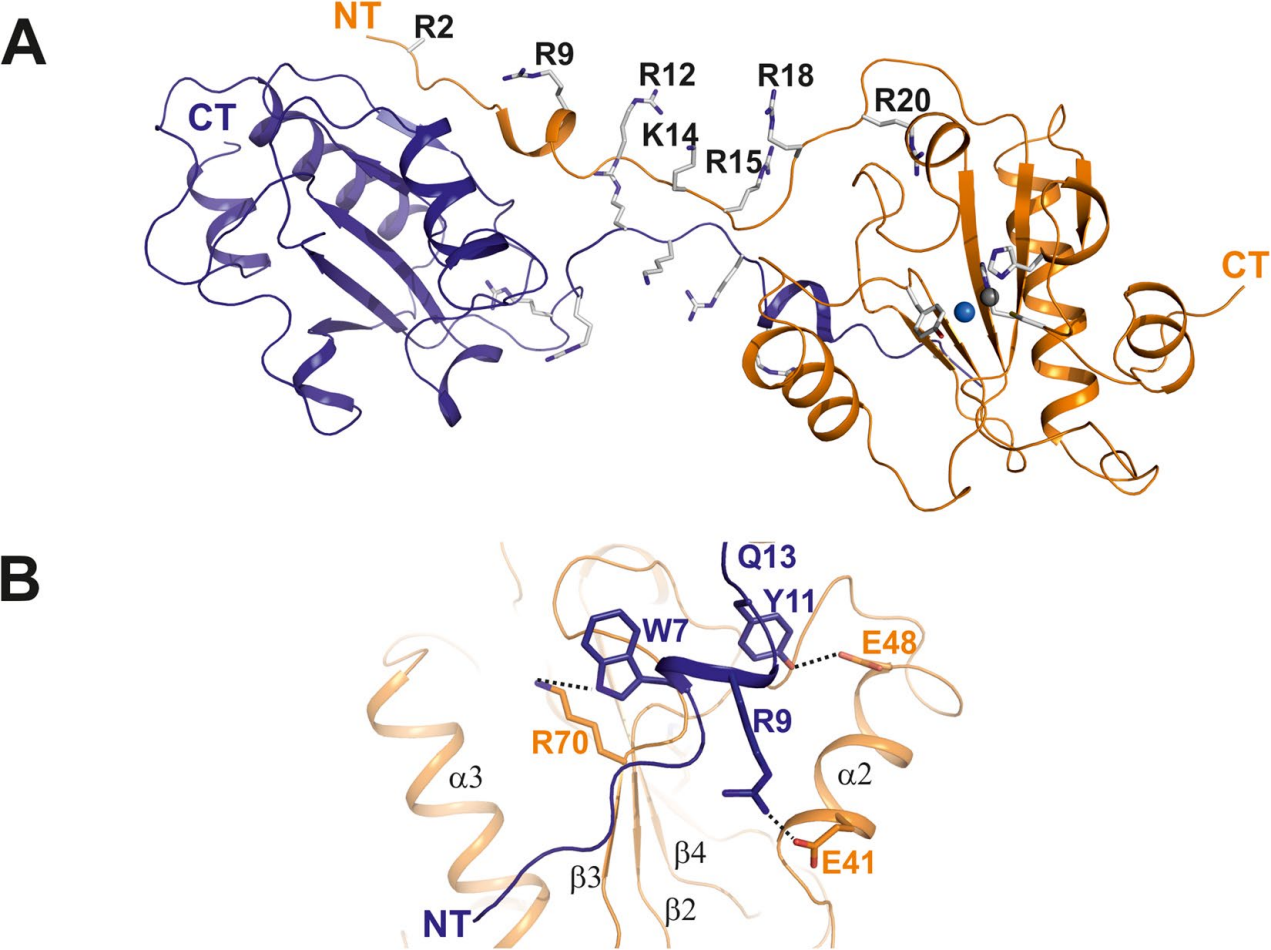
The dimeric structure of Ts2631 endolysin shows a 3D domain swap via the N-terminus. (**A**) Asymmetric unit of Ts2631 endolysin showing two protein molecules, which are highlighted in a ribbon representation. The dimer is interconnected by an elongated and largely unstructured N-terminus (residues 1–20), which carries seven positively charged residues (six arginines, one lysine; shown in stick representation), most of which are pointing towards the same direction (residues in stick representation are denoted by type and numbers). The structure represents a 3D domain swap of two interconnected monomers. (**B**) Molecular interactions between the N-terminus of monomer A (in blue) and the globular domain of monomer B (in orange) are marked by dashed lines. Interacting residues are marked in the same color as their respective domains and numbered according to their position in the sequence.

Ts2631 endolysin crystals in space group P2_1_2_1_2_1_ diffracted to 1.95 Å resolution, and the structure was determined by the molecular replacement method using the coordinates of T7 lysozyme as a search model (PDB entry: 1LBA)^7^. The data collection and refinement statistics are summarized in Table 1. There are two molecules in the asymmetric unit and the Cα positions of these molecules (residues 16–156) superimpose with a low r.m.s.d. (0.6 Å). Only the flexible N-terminal region shows structural variability, which is due to a deviation of the two molecules from perfect twofold non-crystallographic symmetry (Fig. 1). The two monomers in the asymmetric unit interact with each other via the N-terminus and an interaction of the N-terminal extension with the globular domain (Fig. 2A). Initially, we reasoned that this interface might be involved in dimerization, but biochemical data based on analytical ultracentrifugation together with a detailed analysis using PISA software (PISA score: 0.25) pointed towards the monomer as the dominant form in solution (Supplementary Fig. S1)^15^. Given the evolutionary relationship of Ts2631 endolysin with PGRPs, we analyzed their structural similarity to Ts2631 endolysin in more detail. When the dimeric Ts2631 protein (molecules A and B) was superimposed onto proteins with the PGRP fold (PDB entries: 2EAX for human PGRP-I beta and 1SK4 for human PGRP-I alpha), it became clear that the N-terminal extensions indeed form a 3D swap. When the core domain of molecule A (residues 16–156) was superimposed on PGRP, the N-terminus of molecule B superimposed well on the N-terminus of the PGRP protein (Supplementary Fig. S2). Therefore, we constructed a monomeric structure using the globular domain (residues 14–156) of monomer A and the N-terminus of monomer B (residues 1–13) and vice versa (Fig. 2B). Both monomers modeled in this way are identical, and in each monomer, three hydrogen bonds (Trp7′/Lys70″, Arg9′/Glu41″ and Tyr11′/Glu48″) loosely connect the N-terminus to the enzyme core domain (Fig. 2B).

**Table 1:** Data collection and refinement statistics.

	Endolysin
**Data collection**	
Space group	P2_1_2_1_2_1_
**Cell dimensions**	
*a*, *b*, *c* (Å)	53.58, 56.09, 116.72
α, β, γ (°)	90, 90, 90
Resolution (Å)	50–1.95 (2.07–1.95)
*R*_sym_ or *R*_merge_	0.07 (0.98)
CC*	99.9 (70.2)
*I*/*σI*	14.1 (1.32)
Completeness (%)	92.1 (92.1)
Redundancy	4.4 (4.5)
**Refinement**	
Program	PHENIX
Resolution (Å)	50–1.95 (2.02–1.95)
No. reflections	26266 (2719)
*R*_work_/*R*_free_	0.20/0.24 (0.34/0.35)
**No. atoms**	
Protein	603
Water	177
Zinc	2
***B*** **-factors**	
Protein	28.2
Water	42.9
Zinc	81.5
**R.m.s. deviations**	
Bond lengths (Å)	0.03
Bond angles (°)	0.66
**Ramachandran statistics**	
Residues in favored region (%)	95.4
Residues in allowed region (%)	4.6
Residues in outlier region (%)	0
PDB entry	6FHG

*Values in parentheses are for the highest-resolution shell.

### Molecular architecture of the endolysin active site

**Figure 3:**
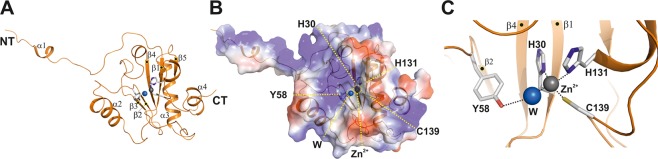
The crystal structure of Ts2631 endolysin shows a conserved peptidoglycan binding site. (**A**) Structure of the globular protein presented in a cartoon representation with the extended N-terminal tail comprising a short α-helix, α1 (marked NT and α1). The secondary structure assignment to the mixed α/β structure is α1/β1/β2/α2/β3/β4/α3/β5. The β-sheet is sandwiched between helices α2 and α4, collectively enclosing the active site with the tetragonally coordinated zinc ion (zinc ion represented by a gray sphere; residues are shown in stick representations) in the center of the structure. (**B**) Surface representation of Ts2631 endolysin demonstrating positively charged patches at the N-terminus and around the active site, which is formed by residues His30, His131, Cys139 and a water molecule (W; blue sphere). (**C**) Expanded view of the active site in the same perspective as in (**A**,**B**), with the active site residues numbered and the zinc ion marked. All structure figures were prepared using PYMOL (www.pymol.org).

The Ts2631 endolysin structure displays a mixed fold of a five-stranded β-sheet (β1-β5) that is flanked by helices α2 and α3, one from each side, yielding a sequential secondary structure sequence of α1/β1/β2/α2/β3/β4/α3/β5/α4 (Fig. 3A). Only the C-terminal helix (α4) does not interact with the central β-sheet, but it is connected to α3 via hydrophobic interactions. The overall protein sequence shows a surplus of nine positive charges, and these charges consequently dominate the surface properties around the putative PGN-binding site and the N-terminal extension (Fig. 3B). The active site is located in the center of the globular fold and is composed of two histidine residues (His30, His131) and Cys139, all of which are buried inside the structure, which has an elongated cleft-like architecture (Fig. 1). His30 is located on β2, while His131 and Cys139 are located on a flexible loop structure connecting β5 with α3 (Fig. 3A,C). Residues on this loop structure of both monomers show higher B-factors and hence higher mobility, which may allow adaptation to substrate binding (Fig. 3C). A water molecule stabilized by the conserved residue Tyr58 completes the tetragonal coordination sphere of Zn^2+^ (Fig. 3C).

### Ts2631 endolysin homologs show variation in the catalytic center

**Figure 4:**
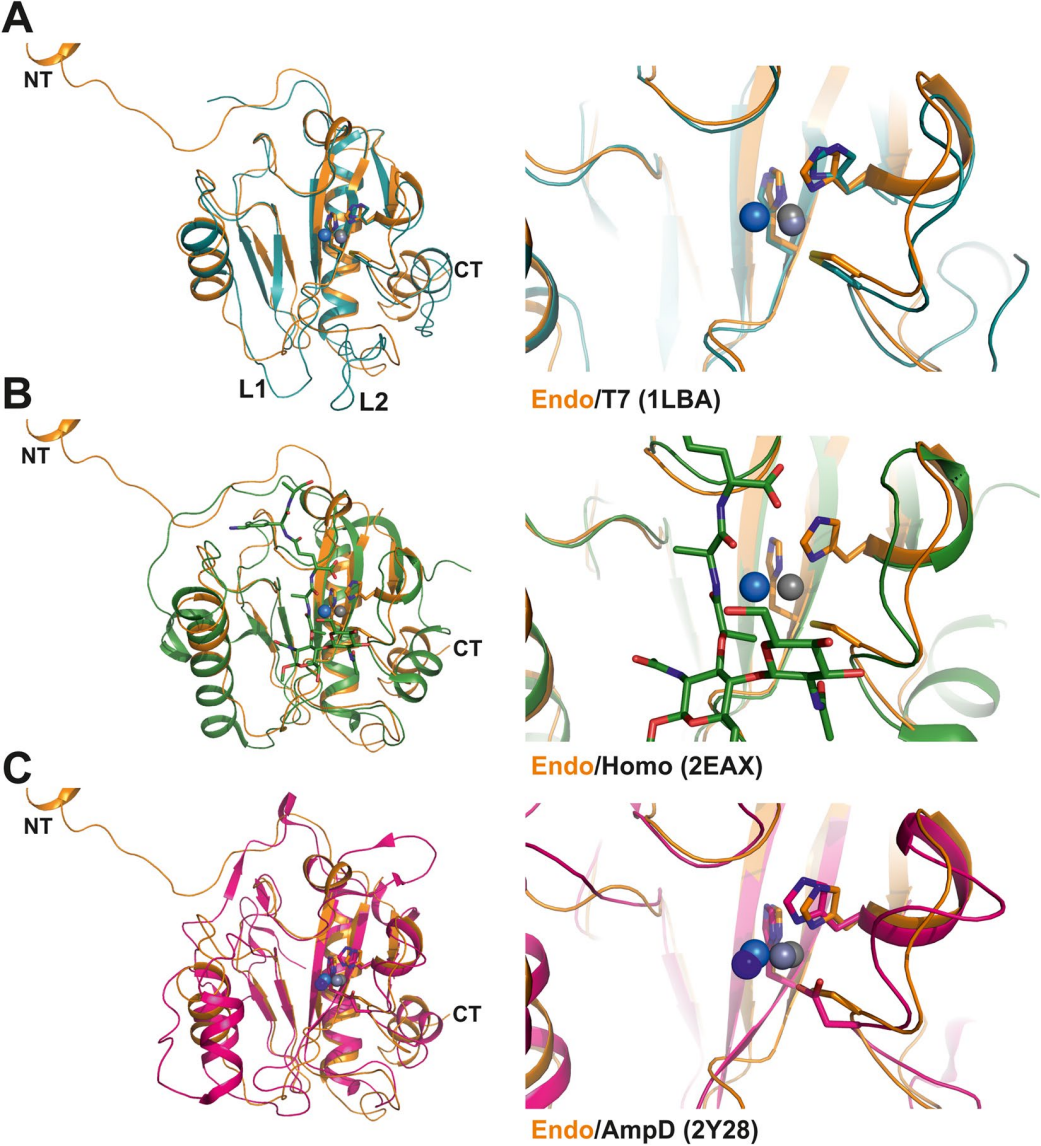
Structural comparison of Ts2631 endolysin homologs. (**A**) Superposition of the phage endolysins Ts2631 (in orange) and T7 lysozyme (in cyan; PDB entry: 1LBA) shown in ribbon representation. The two phage structures show a structural r.m.s.d. of 1.6 Å for 120 aligned Cα atoms and the same active site residues (Fig. on the right). (**B**) Superposition of Ts2631 endolysin with the structural homolog PGRP from *Homo sapiens* (in green; PDB entry: 2EAX; r.m.s.d. of 1.6 Å for 120 aligned Cα atoms. (**C**) Structural comparison of Ts2631 and the bacterial AmpD protein structure from *Citrobacter freundii* (r.m.s.d. 1.9 Å for 125 aligned Cα positions).

The T7 phage lysozyme and human PGLYRP2 possess the same active site residues as Ts2631 endolysin (Fig. 4A and Supplementary Fig. S3). Eukaryotic PGRPs have a similar fold as phage endolysins; however, most PGRPs recognize but do not degrade PGN due to differences between their active site residues and those of phage endolysins (e.g., Cys/Ser, His/Ile; see Supplementary Fig. S3 for a structural alignment of T7-like proteins). The PGRP structures most similar to the Ts2631 endolysin structure are those of the human PGRPs PGRP-Iα and PGRP-Iβ (PDB entries: 1SK4 and 2EAX, respectively) (Fig. 4B; Supplementary Fig. S2). In the superposition models of Ts2631 endolysin and the complex structure of human PGRP, the common substrate N-acetylmuramyl-L-Ala is 2.3 Å distant from the Zn^2+^ ion of the Ts2631 enzyme (Fig. 4B). This distance is ideal for bond activation and hydrolysis, indicating that this structure is conserved between the two scaffolds. In the bacterial autolysin AmpD, the active site is changed to a His/His/Asp triad binding Zn^2+^, where the aspartic acid residue replaces Cys139 of the Ts2631 endolysin (Fig. 3C and Supplementary S3).

### Selection of residues for site-directed mutagenesis

To determine the residues responsible for the lytic and substrate binding activity of the Ts2631 endolysin, we analyzed the structure of human PGRP-Iβ (PDB entry: 2EAX) co-crystallized with a muropeptide^10^. We found that the sugar and peptide moieties of the PGN ligand interact with PGRP-Iβ via Thr241, Tyr274, Asp301, Arg353, and Thr354 (Supplementary Fig. S4A) and that these residues correspond to Thr32, Tyr58, Asn85, Val135 and Thr137 in Ts2631 endolysin, respectively (Supplementary Fig. S4B). Most of these residues are also conserved in the T7 lysozyme, but Thr137 is replaced by a lysine, which is the same difference that is observed in AmpD^8^. *In silico* analysis of the Ts2631 endolysin sequence (https://www.ncbi.nlm.nih.gov/protein/677570412) highlights twelve residues that might be responsible for interactions with the substrate (Supplementary Fig. S3). Those residues are His31, Thr32, Pro54, **Tyr58**, Leu72, Ile79, Asn85, **His131**, Val135, **Thr137**, Glu138 and **Cys139** (residues in boldface type indicate amino acids that are critical for lytic activity). These residues were subjected to further analysis.

We also analyzed the Ts2631 endolysin structure to identify conserved and surface-exposed amino acids that may have an additional effect on protein activity and PGN binding (Supplementary Fig. S5). In total, we constructed the nineteen Ts2631 endolysin single-residue substitution variants listed in Table 2. Previously, we have shown that the activity of five substitution variants forming the catalytic core was significantly less than that of the wild type^5^. Here, these variants (H30N, Y58F, H131N, T137K and C139S), which had been constructed and purified previously^5^, served as a control in an experiment in which we tested the effect of surface-exposed amino acids on the lytic activity of the Ts2631 endolysin.

**Table 2:** Summary of properties of Ts2631 endolysin variants.

No.	Variant	Lytic activity*	Insoluble peptidoglycan binding^§^
1	wt Ts2631	+++	+
**2**	**H30N**	−	+
**3**	**Y58F**	−	+
**4**	**H131N**	−	+
**5**	**T137K**	−	+
**6**	**C139S**	−	+
**7**	***Y60A***	−	−
**8**	***K70A***	−	−
**9**	H31A	−	+
**10**	T32A	−	+
**11**	C80A	−	+
**12**	N85A	−	+
13	A33G	+++	+
14	P54A	+++	+
15	R64A	+++	+
16	D65A	+	+
17	R67A	+++	+
18	Y69A	+++	+
19	L72A	++	+
20	I79A	++	+
21	G95A	+++	+
22	D96A	+++	+
23	N133A	+++	+
24	V135A	++	+
25	E138A	+++	+
26	Δ2-22	+++	+/−

The Ts2631 endolysin variants are grouped according to the effect of their mutation on the function; bold indicates residues responsible for bacteriolytic activity, italic indicates residues essential for peptidoglycan binding, and underline defines residues located in the PGN-binding groove that participate in the substrate binding, as indicated by comparative analysis with eukaryotic PGRPs.

*Lytic activity was estimated by spectrophotometric measurements of the decrease in the turbidity of a chloroform-treated *T. thermophilus* HB8 suspension after the addition of the specified variant: +++ more than 60% activity relative to wild-type Ts2631 endolysin; ++ between 50 and 60%; + between 30 and 40%; − less than 20% or no visible activity.

^§^Insoluble peptidoglycan binding activity was measured by a PGN binding assay: + binding; −no binding; +/− the protein was predominantly in the unbound fraction.

### Lytic activity of Ts2631 endolysin variants

**Figure 5:**
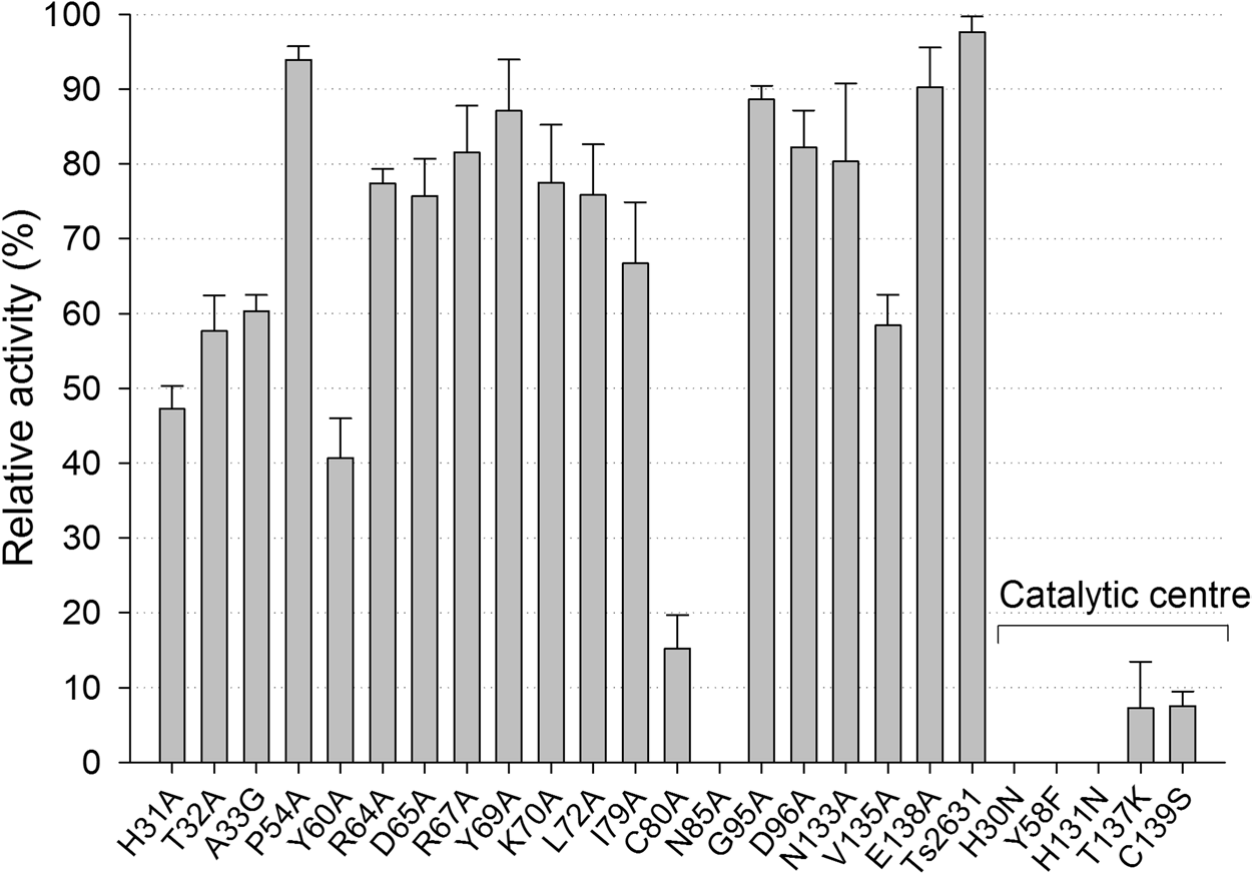
Lytic activity of Ts2631 endolysin mutants at 60 °C against chloroform-treated *T. thermophilus* HB8 substrate. Bacteriolytic activity was measured in a standard turbidity reduction assay in a 96-well plate format. Activities are expressed as percentages relative to the native Ts2631 endolysin activity. Mutations are identified by the one-letter code for the wild-type amino acid followed by the residue number and then the one-letter code for the mutant amino acid. Error bars indicate the standard deviation (n = 3).

We used a turbidity reduction assay (TRA) to spectrophotometrically measure decreases in the turbidity of suspensions of chloroform-treated (outer membrane-permeabilized) *T. thermophilus* cells at 60 °C after the addition of selected endolysin variants. For most substitution variants, the lytic activity exceeded 50% that of the wild-type protein (Fig. 5, Table 2). The activity of the N85A mutant was undetectable by this assay under the conditions that were used. Moreover, a high reduction in the activity of the C80A variant was observed, remaining at an average level of 15% (Fig. 5). Both variants were soluble, with no signs of aggregation and significant structural changes as evaluated by circular dichroism spectrum analysis (Supplementary Fig. S6). This result excluded the possibility that improper protein folding had an impact on the lytic function of C80A and N85A variants.

**Figure 6:**
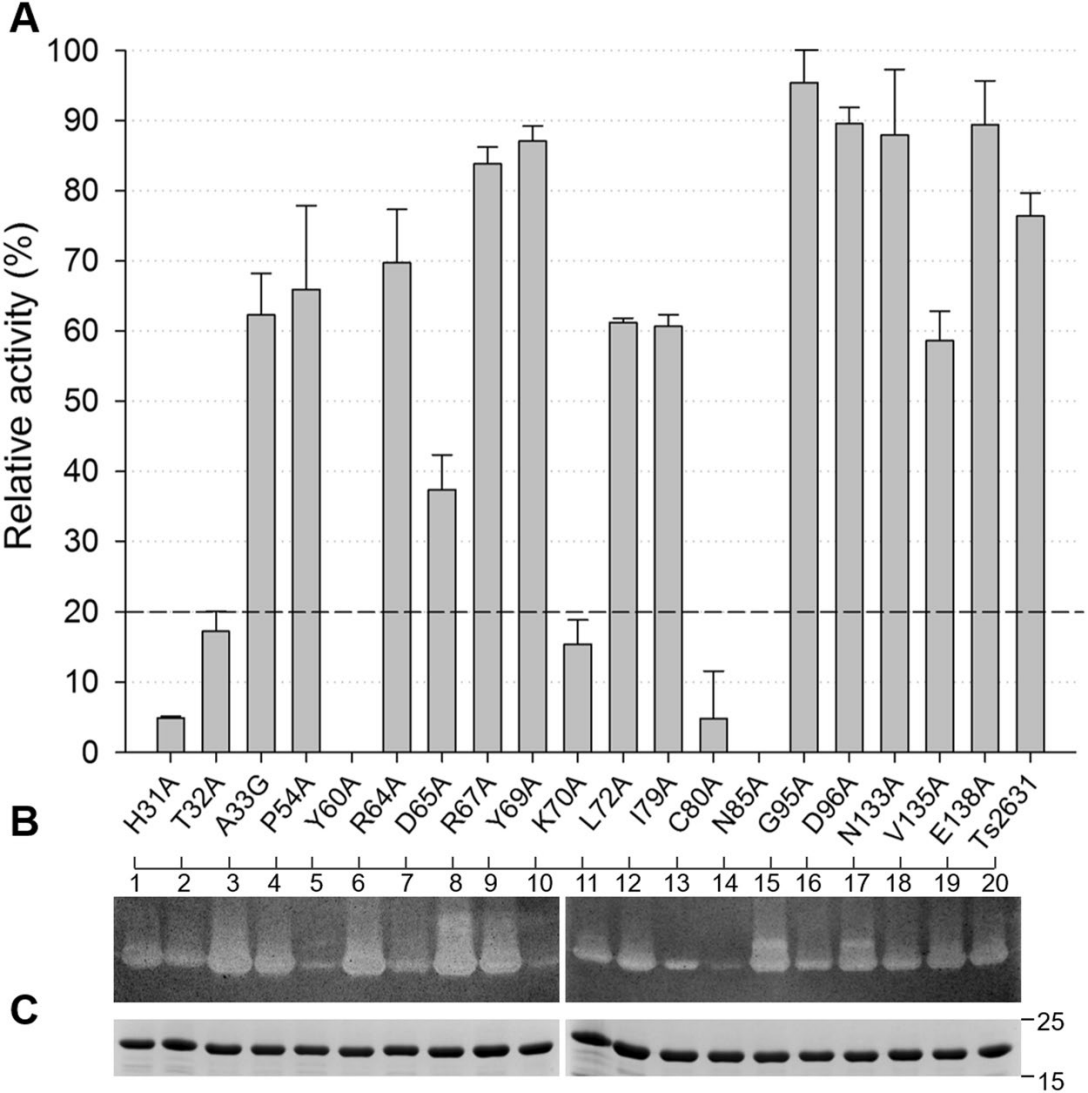
Lytic activity of Ts2631 endolysin variants at 37 °C. (**A**) Bacteriolytic activity was measured in a standard turbidity assay against chloroform-treated *T. thermophilus* HB8 substrate at 37 °C. Activities are expressed as percentages relative to the maximal lytic activity achieved among the dataset (note that the activity of native Ts2631 endolysin is not 100% under these experimental conditions). The dark gray dashed line represents 20% activity. Error bars indicate the standard deviation (n = 3). (**B**) Zymogram analysis of purified Ts2631 endolysin variants. Numbers 1–10 correspond to H31A, T32A, A33G, P54A, Y60A, R64A, D65A, R67A, Y69A, K70A (first gel), respectively, and numbers 11–20 indicate L72A, I79A, C80A, N85A, G95A, D96A, N133A, V135A, E138A and native Ts2631 endolysins (second gel). (**C**) SDS-PAGE of purified Ts2631 endolysin variants (0.5 µg) loaded on two separate gels served as a loading control for a zymogram assay. Molecular weight markers are shown on the right.

To our surprise, the substitution of many conserved amino acids had no effect on the enzyme lytic activity at 60 °C. Therefore, we decided to lower the reaction temperature to 37 °C, slowing the reaction and potentially allowing the observation of more pronounced differences between the reactivity of Ts2631 endolysin variants. At 37 °C, we noticed no lytic activity in case of the Y60A mutant and reduced activity by three other variants: H31A, T32A and K70A, which reached no more than 17.2% of the activity of the wild-type enzyme (Fig. 6A). These results were further validated by a zymogram technique, which is not a quantitative method but is more sensitive than the TRA (Fig. 6B, whole gels in Supplementary Fig. S9A). Zymograms showed a pattern of lytic activity of Ts2631 endolysin variants that was similar to that provided by the TRA at 37 °C. Polyacrylamide gel electrophoresis of purified endolysin and its variants served as a loading control, showing that equal amount of proteins were subjected to the analysis (Fig. 6C, whole gels in Supplementary Fig. S9B). Residues His31, Thr32, Cys80 and Asn85 are situated in the PGN-binding groove of Ts2631 endolysin (Supplementary Fig. S4B).

### Peptidoglycan binding tests of Ts2631 endolysin variants

**Figure 7:**
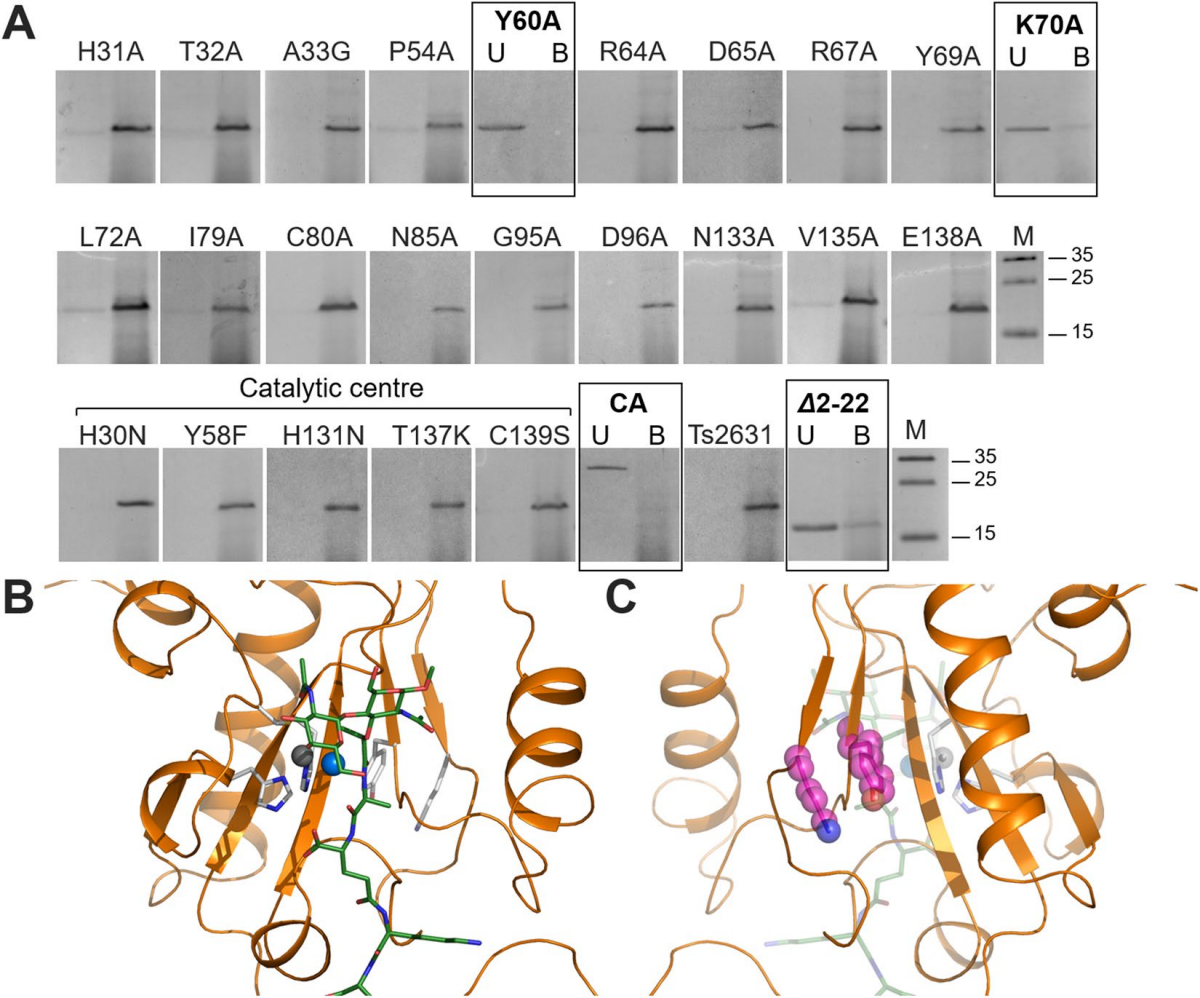
Evaluation of peptidoglycan binding activity of the Ts2631 endolysin variants. (**A**) Ts2631 endolysin variants were mixed with purified *T. thermophilus* HB8 peptidoglycan, and the proteins remaining in the supernatant (*U*, unbound) or bound to peptidoglycan (*B*, bound) were separated by centrifugation at 12,600 × g for 5 min at 4 °C. Proteins were analyzed by SDS-PAGE. Wild-type Ts2631 endolysin and carbonic anhydrase (CA) were used as positive and negative controls, respectively. Frames indicate proteins predominant in the supernatant fraction, namely, Y60A, K70A, the negative control (CA) and the Ts2631 endolysin truncated derivative Δ2-22. M - PageRuler prestained protein ladder, 10 to 180 kDa (Thermo Fisher Scientific). (**B**) The front side of the PGN-binding groove. (**C**) The back side of the PGN-binding groove with residues Tyr60 and Lys70 highlighted in pink.

To go one step further in our analysis of the Ts2631 endolysin ability to bind PGN, we applied a methodology developed previously by another research group^9^. This method was routinely used in the case of wild-type PGRPs and was used here for the first time to investigate the binding of the endolysin and its substitution variants to a large PGN polymer. We purified the PGN of *T. thermophilus* HB8 and performed protein-substrate binding assays of all Ts2631 endolysin variants. Wild-type Ts2631 endolysin was used as a positive control, and carbonic anhydrase from bovine erythrocytes (29 kDa) served as a negative control. Out of the 24 tested single-substitution variants of Ts2631 endolysin, only two, Y60A and K70A, did not bind the purified and insoluble PGN. Both Y60A and K70A were predominantly not bound to PGN and thus present in the supernatant fraction (Fig. 7A, Table 2, selected full-length gels are in Supplementary Fig. S10). The crystal structure data show that the two residues lie in close proximity to each other but opposite to the PGN-binding cleft (Fig. 7B,C). These results are the first to show the endolysin surface residues, other than the residues present in the PGN-binding groove, that play a role in substrate binding.

### Function of the N-terminal extension in peptidoglycan binding

**Figure 8:**
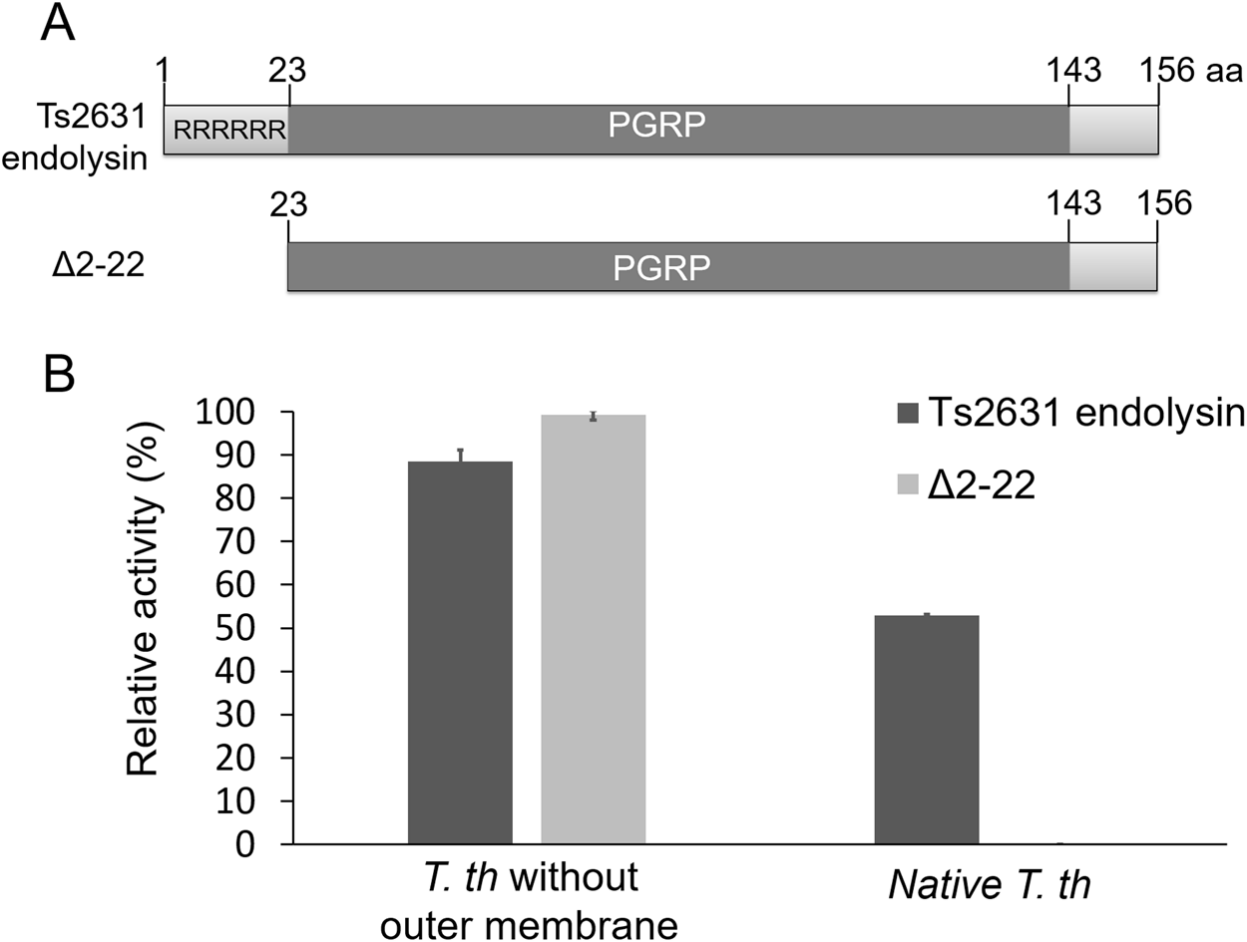
Activity of the full-length and deletion derivative of Ts2631 endolysin. (**A**) The schematic maps of the full-length and deletion derivative of Ts2631 endolysin. RRRRRR – N-terminal arginine rich region. PGRP – peptidoglycan recognition domain (also known as type 2 amidase domain). (**B**) Mutant activity was measured in a standard turbidity assay against chloroform-treated and intact *T. thermophilus* HB8 substrate at 60 °C. Activities are expressed as percentages relative to the maximal lytic activity achieved for Δ2-22 variant. Error bars indicate the standard deviation (n = 3).

Homology searches for the Ts2631 endolysin N-terminal extension (residues 1–20) using the T-Coffee computer program^16^ have shown that it does not share amino acid sequence identity with any particular sequence in databases (Supplementary Fig. S7, sequence data for the phage database; data for the entire sequence database is not shown). Nevertheless, this N-terminal extension is positively charged, making it similar to polycationic antibacterial peptides, which kill bacteria by forming pores in the cell membrane^17^. To investigate the role of this unique N-terminal extension, we constructed a deletion variant of Ts2631 lacking amino acids 2–29 (Ts2631Δ2-29). Unfortunately, attempts to purify Ts2631Δ2-29 were ineffective due to the aggregation of the protein. Consequently, a shorter variant missing residues 2-22 was constructed and successfully purified (Fig. 8A). This Ts2631Δ2-22 variant was fully active at 60 °C towards chloroform-treated cells (Table 2). To further analyze the importance of the N-terminal extension in substrate anchoring, we used the PGN binding assays described above. The results showed that Ts2631Δ2-22 was present in both the PGN-unbound and PGN-bound fractions, suggesting a role of the N-terminal extension in binding Ts2631 endolysin to its substrate (Fig. 7A). In a parallel experiment, we tested the ability of Ts2631 endolysin to lyse intact bacteria that had not been treated with chloroform. All previous experiments were carried out with bacteria that had their outer membrane permeabilized by treatment with chloroform-Tris-HCl to expose PGN^18^. Upon the addition of the wild-type endolysin to intact *T. thermophilus* cells, we observed a reduction in the turbidity of the cell suspension, indicating the antibacterial role of Ts2631 endolysin (Fig. 8B). In contrast, the Ts2631Δ2-22 variant fully active against outer membrane permeabilized cells (100% activity) was not functional in the presence of intact *T. thermophilus* HB8 and showed no signs of lysis after extended (3 h) incubation (Fig. 8B). This result suggests that the N-terminal extension allows endolysin to pass through the outer membrane and then interact with the PGN layer.

## Discussion

Studies on novel endolysins are important for the development of effective antimicrobial agents as alternative to traditional antibiotics^2,19,20^. The Ts2631 endolysin is the first lytic enzyme from a thermophilic bacteriophage whose structure has been determined. The catalytic core is formed by His30, His131 and Cys139, a catalytic motif also present in the T7 lysozyme molecule^7^. The crystal structure data are also supported by our previous study in which we have shown that modification of catalytic core residues substantially reduces the Ts2631 endolysin lytic activity^5^.

**Figure 9:**
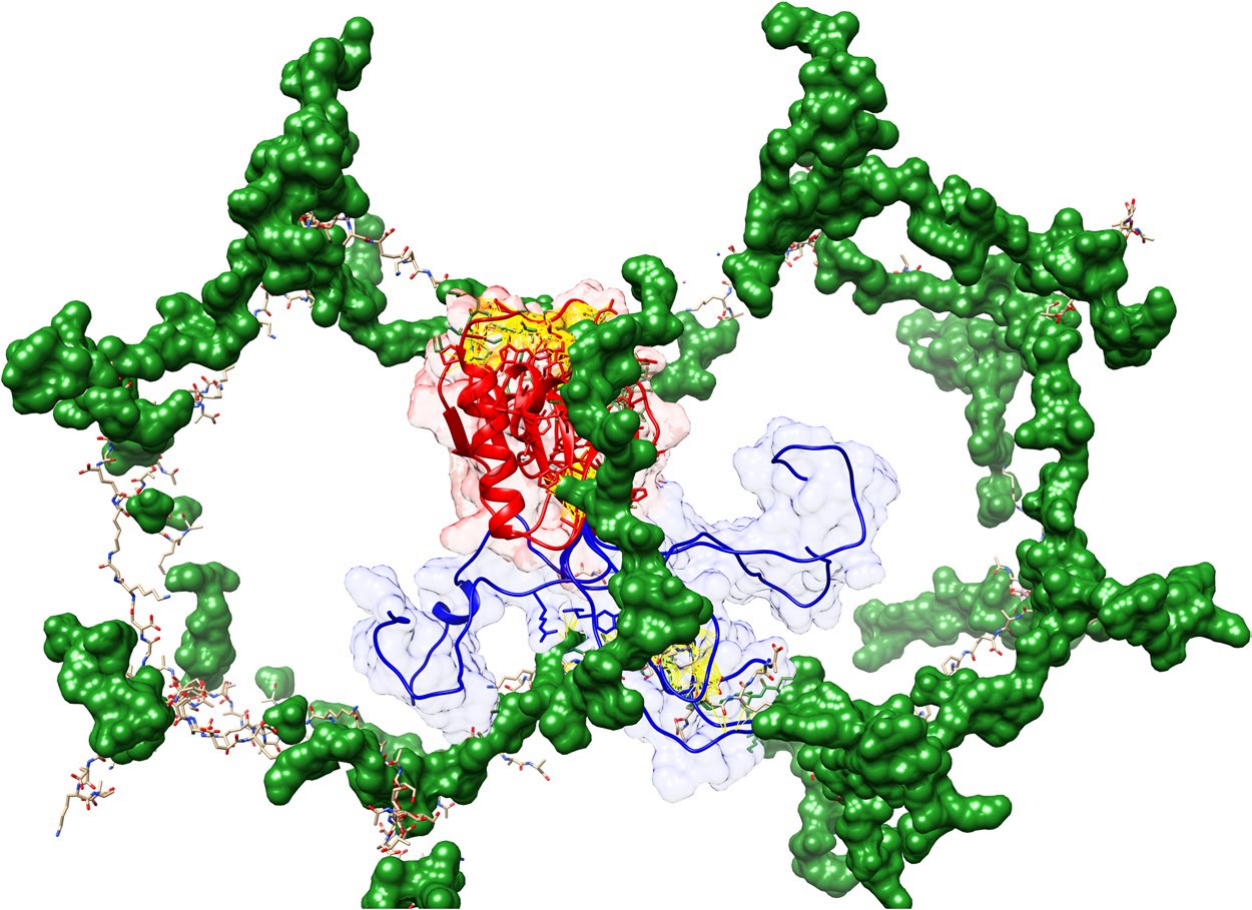
Model of Ts2631 endolysin binding to peptidoglycan. The Ts2631 endolysin consist two major parts: the structural part with catalytic residues (in red) and flexible N-terminal extension (in blue). Both parts are essential for peptidoglycan (in green) binding (the possible contacts are marked in yellow). While structural part is responsible for lysis of peptidoglycan (coordinated by Zn^2+^ and water molecule and highly conserved catalytic triad), the N-terminal extension can adopt multiple conformations with putative contacts with peptidoglycan far away from the main body of the enzyme (the alternate conformers of the N-terminal extension had been built using Modeller software with DOPE potential)^21,22^.

In contrast to T7 lysozyme, Ts2631 endolysin contains a unique N-terminal sequence that is not found in the phage homologs. Crystallographic and *in silico* data indicate that this positively charged N-terminal extension of Ts2631 endolysin is flexible and may assume alternative conformations, as depicted by loop modeling via the Modeller program (Fig. 9)^21,22^. This spatial conformation suggests that the N-terminal extension interacts with negatively charged bacterial membranes and PGN. Interestingly, the eukaryotic PGRPs also have the N-terminal PGRP-specific region^11^, which is usually hydrophobic, varies among these proteins and shows no amino acid sequence resemblance to Ts2631 endolysin^5^. However, similarly to the N-terminal extension of Ts2631 endolysin, the N-terminal region comprising residues 12–33 of the *Drosophila* amidase PGRP-LB was shown by crystallography to be located at the back of the active site^12^. The role of this region in PGRP-LB is still unknown, although its function in binding additional ligands has been proposed but not supported by experimental data^12^. Thus, our experiments with a deletion variant of Ts2631 endolysin deprived of the N-terminal extension may shed some light on the general function of the N-terminal region.

Unique among lytic enzymes is also a short polyglycine stretch of four consecutive residues at the C-terminus of Ts2631 endolysin (Supplementary Fig. S7). The only protein with this polyglycine track in the literature was the precursor of the protein translocation channel of the outer envelope membrane of chloroplasts. There, the polyglycine track was shown to be necessary for directing the protein to the chloroplast outer membrane^23^. We hypothesize that by analogy, the four-glycine segment of Ts2631 endolysin might be employed to target bacterial membranes.

The crystal structure, supported by biochemical data, helped us to reveal the substrate-binding location of Ts2631 endolysin. His31 and Thr32 are part of the HHT motif characteristic for PGRPs^5,19^. The residue Cys80 is conserved in 94% of all insect and mammalian PGRPs and is required for their proper conformation^24^. Surprisingly, this cysteine is not preserved in the phage T7 lysozyme^7^. Asn85 is also conserved among PGRPs, and its role in accommodating the fourth D-Ala residue of the PGN stem peptide was previously proposed for PGRP-IαC^25^. Therefore, by analogy to PGRPs, whose corresponding residues are located in the PGN-binding cleft, we propose a role of the above-mentioned residues in substrate binding.

In addition, two other residues, Tyr60 and Lys70, are essential for PGN interactions. Both of these residues are present in the primary sequences of Ts2631 and Ph2119 endolysins derived from *Thermus scotoductus* thermophilic phages but are not conserved among mesophilic PGRPs (Supplementary Fig. S7). Their location at the back of the PGN-binding groove was surprising, as the literature search did not provide any examples resembling this kind of protein spatial architecture. The binding to the PGN may be explained by possible CH-π interactions between aromatic residues (such as Tyr) and the glycan strands of the PGN. The interaction may be further stabilized by hydrogen bonds via a water molecule between glycan and Lys^26,27^. Therefore, both Tyr60 and Lys70 may have roles in binding to the glycan moiety.

The general pattern of interaction between the endolysin molecule and the PGN mesh remains unknown. The perpendicular model of the bacterial cell wall visualizes PGN as a honeycomb^28^. It was previously proposed for the human PGRP-IβC that the protein is located in a ≈120 Å in diameter, incomplete pore of PGN formed in growing cell walls^10^. In that model, PGRP-IβC interacts with PGN by its PGN-binding groove while the opposite site of the molecule remains unconnected to the substrate. In agreement with that pattern, Ts2631 endolysin residues His31, Thr32, Cys80 and Asn85, which are located in the PGN-binding groove, may be responsible for interacting with the substrate. Moreover, based on our experimental data, we propose a scenario in which Tyr60 and Lys70—the residues situated opposite to the active site—also dock the protein within PGN pores. Both residues are present in the Ts2631 Δ2-22, therefore, the variant without the N-terminal region can maintain partial PGN binding.

We stipulate that this arrangement might be an adaptation by thermophilic phage endolysins to function at elevated temperatures, where stronger docking of endolysin to the PGN would improve the overall performance of the lytic enzyme.

The unusual thermostability (a melting temperature of 99.8 °C) is a striking feature of Ts2631 endolysin^5^, although, on average, proteins derived from thermophiles have a melting temperature 31.5 °C higher than that of their mesophilic homologs^29^. The current view is that enzyme adaptations made to withstand high temperature involve increased compactness of protein hydrophobic core, metal ion binding, replacing polar residues with charged residues, shortening surface-exposed loops, increasing the number of disulfide bonds and forming salt bridges between pairs of charged residues^30^. To elucidate the structural determinants of Ts2631 endolysin thermostability and temperature-dependent activity, we analyzed the endolysin amino acid sequence. Factor that might contribute to the stabilization of Ts2631 endolysin is the formation of extended hydrophobic cores by the high number of tryptophans (4.49%) (Supplementary Fig. S8A). In mesophiles, the percentage of tryptophans on average does not exceed 1.27%^31^. Trp residues in well-buried states are known to participate in cation-π interactions that maintain the conformational stability of protein structures^32^, and a higher frequency of cation-π interactions in well-buried states might be correlated with protein thermostability^33^. Moreover, the percentage of prolines (8.5%) is clearly greater than the average content of prolines in mesophilic proteins (6.3%)^34^. These residues are evenly distributed throughout the protein (Supplementary Fig. S8B) and locally lower the B-factors, indicating their stabilizing effect. It has been postulated that abundance of prolines and small-volume residues may minimize hindrance and entropy expenditure due to the side chain burial, thus favoring looping and bending in proteins and leading to the formation of compact core regions in thermophilic protein structures^35^. Finally, arginine residues are also implicated in thermal stability due to their guanidinium-like head group, which can have various interactions with ions^36^. These features are clearly distinct from those of mesophilic phage endolysins and may explain the observed thermostability of proteins such as the Ts2631 endolysin. Obviously experimental data are necessary to fully understand the thermal stability pattern of Ts2631 endolysin and such analysis will be carried out within the framework of a future project.

As thermostable proteins are superior in medical and technical processes^37–39^ due to their rigid structure and protease resistance, thermostability is a desired feature of any novel enzyme considered for potential applications. Ts2631 endolysin fulfills this requirement. Additionally, it not only is an extremely stable phage endolysin but also carries a natural Arg-rich N-terminal extension, which may help the protein interact with bacterial membranes when applied as an external antimicrobial agent.

## Materials and Methods

### Bacterial strains and growth conditions

Chemically competent *Escherichia coli* DH5α and BL21(DE3) cells (Invitrogen) were prepared for site-directed mutagenesis and recombinant protein expression, respectively. These bacteria were cultivated at 37 °C in Luria Broth (LB) medium with shaking. *T. thermophilus* HB8 was cultured as previously described^5^. The plasmid pLT1 (a derivative of the pET15b vector) was used for the overproduction of Ts2631 endolysin and served as a template for site-directed mutagenesis studies of Ts2631 endolysin^5^. The plasmid pRARE (Cm^R^) served as a source of tRNAs for *E. coli* rare codons (Novagen) to compensate for codon usage bias. When necessary, media were supplemented with 100 µg ml^−1^ of ampicillin (Ap) and 30 µg ml^−1^ of chloramphenicol (Cm). The plasmid pLT1, overexpressing the Ts2631 endolysin gene, as well as plasmids used for the overproduction of the Ts2631 endolysin variants constructed in the present study, were deposited in the Collection of Plasmids and Microorganisms, University of Gdansk, Poland (KPD, World Data Center for Microorganisms registered no. 1084).

### Protein overproduction and purification

*E. coli* BL21(DE3)[pRARE] cells carrying the expression plasmids were cultivated at 37 °C in 500 ml of LB to an OD_600_ of 0.4–0.5. Overproduction of Ts2631 endolysin and its derivatives was induced with 1 mM isopropyl-β-D- thiogalactopyranoside (IPTG) for 4 h at 37 °C. The cells were subsequently harvested by centrifugation (10,000 × g for 20 min, 4 °C). The cell pellet was resuspended in 30 ml of NPi buffer (50 mM NaH_2_PO_4_, pH 8.0, 300 mM NaCl, 10 mM imidazole, 0.1% Triton X-100, 10% [vol/vol] glycerol, 2 mM β-mercaptoethanol, and 1 mM phenylmethylsulfonyl fluoride [PMSF]) and disrupted by sonication (30 bursts of 10 s at an amplitude of 12 μm). The cellular debris was removed by centrifugation (10,000 × g for 20 min, 4 °C), and overproduced proteins were purified from clarified supernatants on TALON cobalt metal affinity resin according to the manufacturer’s procedure for bath/gravity-flow column purification (Clontech Laboratories, Inc., USA). Proteins bound to the TALON resin were eluted with 150 mM imidazole in NPi buffer, and pooled fractions containing pure proteins (as judged by SDS-PAGE) were dialyzed against D buffer (25 mM potassium phosphate buffer, pH 8.0, 50 mM KCl, 0.1% Triton X-100, 50% glycerol, and 0.1 mM ZnSO_4_). For crystallography studies, the Ts2631 endolysin was dialyzed against 20 mM MES buffer, pH 6.0. The Bradford assay was used to determine the protein concentration^40^, and purified proteins were stored at −80 °C until further analysis.

### Crystallization, data collection and structural analysis

Protein at a concentration of 8 mg ml^−1^ was applied to commercial screens from Hampton Research, which yielded crystals under conditions of 20% PEG 3350 and 0.2 mM disodium tartrate. The crystals were mounted using the reservoir conditions with an additional 20% PEG 400 for cryo-protection. Crystals were flash frozen in liquid nitrogen, and data were collected at the ALBA synchrotron at 100 K. Diffraction data were recorded on a PILATUS 6 M detector, and spots were integrated and scaled with the XDS/XSCALE software package. Molecular replacement was performed with Phaser, which was included in the PHENIX program package^41^, and the structure was refined in PHENIX and rebuilt in Coot^42^. The final R/R_free_-values are 20 and 24%, respectively (Table 1).

### Site-directed mutagenesis

Ts2631 endolysin residue A33 was substituted with glycine, resulting in the A33G variant. Eighteen residues (H31, T32, P54, Y60, R64, D65, R67, Y69, K70, L72, I79, C80, N85, G95, D96, N133, V135 and E138) of Ts2631 endolysin were individually substituted with alanine. Ts2631 endolysin variants (H30N, Y58F, H131N, T137K and C139S) were constructed previously^5^ and used herein for lytic activity comparison studies. Site-directed mutagenesis was performed according to the QuikChange II Site-Directed Mutagenesis Kit manual (Agilent Technologies). Primers used in this study are listed in Supplementary Table S1. All constructs were verified by DNA sequencing before being introduced into the *E. coli* BL21(DE3)[pRARE] expression strain by chemical transformation.

### Lytic activity of Ts2631 endolysin

For turbidity reaction assays, the outer membrane of Gram-negative *T. thermophilus* HB8 bacteria was permeabilized by chloroform treatment, as described previously^18^. Briefly, bacteria were grown until late-exponential phase and then centrifuged (4,500 × g, 15 min, 4 °C). The outer membranes of sedimented cells were permeabilized by gentle shaking with chloroform-saturated 50 mM Tris-HCl, pH 7.7 for 45 min at room temperature. The permeabilized cells were then washed, resuspended in 10 mM phosphate buffer (pH 8.0) and adjusted to OD_600_ = 1.0. The turbidity reaction assays were performed in a 96 well plate format by measuring the OD_600_ drop over time (with 0.5 min intervals up to 20 min) in an EnSpire multimode plate reader (PerkinElmer). Each reaction was conducted in triplicate and consisted of 190 μl of permeabilized cells and 10 μl of tested proteins (final concentration 25 μl ml^−1^). The values of negative controls with reaction buffer instead of endolysin were subtracted from the sample measurements. The lytic activity was calculated as follows: [ΔOD_600_ sample (endolysin added) − ΔOD600 (buffer only)]/initial OD_600_. Assays with intact bacteria were conducted as described above, but the permeabilization step was omitted in the procedure. Zymogram qualitative assays were performed with the use of 12.5% SDS-PAGE; the separating gel contained 0.2% (wt/vol) chloroform-treated *T. thermophilus* HB8 as a substrate. Ts2631 endolysin and its derivatives were mixed with 2× Laemmli buffer (Bio-Rad) and loaded into the gel. After electrophoresis, the gel was gently shaken at 37 °C for 16 h in a solution of 25 mM phosphate buffer, pH 8.0, and 1% Triton X-100, washed once with water and then stained for 0.5 h with 1% methylene blue in 0.01% KOH. After washing the gel in ultrapure water, the lytic activity of individual protein bands was observed as a clear zone.

### Peptidoglycan isolation

PGN was extracted from *T. thermophilus* HB8 cells by following a method described previously^43^ with a few modifications. Briefly, *T. thermophilus* HB8 cells were grown in a 500 ml flask at 60 °C to an OD_600_ of 0.7–0.8. After centrifugation (5,000 × g, 10 min, room temperature), the cell pellet was suspended in 3 ml of 1× phosphate-buffered saline (PBS). The solution was transferred to 50 ml tubes with 6 ml of 6% sodium dodecyl sulfate (SDS) and then placed in a boiling water bath. After 3 h of boiling, the water bath heat source was turned off, and the mixture was left overnight to slowly cool to room temperature with stirring at 500 rpm. The next day, to remove SDS, the suspension was centrifuged (40,000 × g, 20 min, room temperature), and the pellet was suspended in ultrapure water. Centrifugation/washing steps were repeated three times, and in the last cycle, the pellet was suspended in a solution of 900 µl of 10 mM Tris-HCl, pH 7.2, and 0.06% w/v NaCl. Pronase E (100 µg ml^−1^ final concentration) was added to the sample, and the mixture was incubated at 60 °C for 2 h. Pronase E digestion was stopped by adding 200 µl of 6% SDS and boiling the sample (100 °C, 30 min). To remove SDS, the suspension was subjected to three centrifugation/washing steps, as described above. In the last centrifugation/washing step, the sample was resuspended in 200 µl of 50 mM sodium phosphate buffer (pH 4.9), frozen in liquid nitrogen and left at −80 °C for further analysis.

### Peptidoglycan binding assay

The PGN binding assays were carried out as described previously^9^. Briefly, 40 μl of purified proteins (Ts2631 endolysin substitution variants, wild-type Ts2631 endolysin and deletion mutant Ts2631Δ2-22) at a concentration of 90 μg ml^−1^ was mixed with 120 μl of T-M buffer (10 mM Tris maleate buffer, pH 6.5, 0.15 M NaCl) and 160 μl of the PGN washed and suspended in T-M buffer (190 µg per sample) and then incubated at 4 °C for 30 min. The mixtures were then centrifuged at 12,600 × g for 5 min at 4 °C, the supernatants were kept for analysis, and the pellets were washed four times with 300 μl of T-M buffer with 1 M NaCl to remove any residual unbound proteins. After a final centrifugation, washed PGN was extracted with 80 μl of a solution of 62.5 mM Tris-HCl, pH 6.8, 2% SDS, 19% glycerol, and 5% β-mercaptoethanol. The supernatant (20 μl) and extract (20 μl) of the sedimented PGN were subjected to Tricine-SDS-Page and stained with Coomassie brilliant blue. In the pilot experiments, bovine serum albumin (66.46 kDa; Sigma-Aldrich) was successfully applied as a negative control, but for the PGN binding assay, we decided to use protein with a similar molecular weight (the *M*w of Ts2631 endolysin is 20.8 kDa; Supplementary Fig. S1). Therefore, a carbonic anhydrase (EC 4.2.1.1) from bovine erythrocytes (29 kDa; SERVA) was used as a negative control.

### Analytical ultracentrifugation

Sedimentation velocity experiments were performed in a Beckman-Coulter ProteomeLab XL-I analytical ultracentrifuge (Fullerton, USA) equipped with AN-60 Ti 4-hole rotor and 12 mm path length, double-sector charcoal-Epon cells loaded with 400 μl of sample and 410 μl of buffer (20 mM MES, pH 6.0, 150 mM NaCl). The experiments were carried out at 4 °C and 50,000 rpm using continuous scan mode and a radial spacing of 0.003 cm. Scans were collected in 5 min intervals at 280 nm. To verify that dimeric structures were formed not only in crystal but also in solution, we investigated the possibility of concentration-dependent oligomerization. To test that, three different protein samples were used: 1.10 OD, 0.62 OD, and 0.34 OD (at 280 nm) for a 1.2 cm path length. The fitting of absorbance versus cell radius data was performed using SEDFIT software, version 14.7^44^ and a continuous sedimentation coefficient distribution c(s) model covering the range from 0.0–4S. The confidence level was set to 0.68. The biophysical parameters of the buffer, the density and viscosity at 4 °C and the protein partial specific volume (V-bar) were estimated using SEDNTERP software (version 1.09, http://www.jphilo.mailway.com/download.htm). The value of the protein V-bar was calculated to be 0.726 cm^3^ g^−1^ (4 °C).

### Circular dichroism spectra

Circular dichroism spectra were recorded using a JASCO J-815 spectropolarimeter with a path length of 1 mm. Proteins were dissolved in 10 mM potassium phosphate buffer, pH 8.0, and 150 mM ammonium sulfate at a final concentration of 0.15 mM. Each circular dichroism spectrum represents the average of six scans.

## Supplementary information

Supplementary Information

## Data Availability

All data generated or analyzed during this study are included in this article (and its Supplementary Information Files). Accession number for nucleotide sequence of Ts2631 endolysin is KJ561354. PDB entries for Ts2631 endolysin, T7 lysozyme, PGRP-I alpha and PGRP-I beta are 6FHG, 1LBA, 1SK4 and 2EAX, respectively.

## References

[1] Young R (2014). Phage lysis: three steps, three choices, one outcome. J Microbiol.

[2] Nelson DC (2012). Endolysins as antimicrobials. Adv Virus Res.

[3] Chakravorty D, Khan MF, Patra S (2017). Multifactorial level of extremostability of proteins: can they be exploited for protein engineering?. Extremophiles.

[4] Dumorné K, Córdova DC, Astorga-Eló M, Renganathan P (2017). Extremozymes: A Potential Source for Industrial Applications. J Microbiol Biotechnol.

[5] Plotka M (2015). Biochemical Characterization and Validation of a Catalytic Site of a Highly Thermostable Ts2631 Endolysin from the *Thermus scotoductus* Phage vB_Tsc2631. PLoS One.

[6] Sawle L, Ghosh K (2011). How do thermophilic proteins and proteomes withstand high temperature?. Biophys J.

[7] Cheng X, Zhang X, Pflugrath JW, Studier FW (1994). The structure of bacteriophage T7 lysozyme, a zinc amidase and an inhibitor of T7 RNA polymerase. Proc Natl Acad Sci USA.

[8] Carrasco-López C (2011). Crystal structures of bacterial peptidoglycan amidase AmpD and an unprecedented activation mechanism. J Biol Chem.

[9] Yoshida H, Kinoshita K, Ashida M (1996). Purification of a peptidoglycan recognition protein from hemolymph of the silkworm, *Bombyx mori*. J Biol Chem.

[10] Cho S (2007). Structural insights into the bactericidal mechanism of human peptidoglycan recognition proteins. Proc Natl Acad Sci USA.

[11] Royet J, Gupta D, Dziarski R (2011). Peptidoglycan recognition proteins: modulators of the microbiome and inflammation. Nat Rev Immunol.

[12] Kim MS, Byun M, Oh BH (2003). Crystal structure of peptidoglycan recognition protein LB from *Drosophila melanogaster*. Nat Immunol.

[13] Guan R (2005). Crystal structure of a peptidoglycan recognition protein (PGRP) in complex with a muramyl tripeptide from Gram-positive bacteria. J Endotoxin Res.

[14] Leone P (2008). Crystal structure of *Drosophila* PGRP-SD suggests binding to DAP-type but not lysine-type peptidoglycan. Mol Immunol.

[15] Krissinel E, Henrick K (2007). Inference of macromolecular assemblies from crystalline state. J Mol Biol.

[16] Di Tommaso P (2011). T-Coffee: a web server for the multiple sequence alignment of protein and RNA sequences using structural information and homology extension. Nucleic Acids Res.

[17] Agier J, Efenberger M, Brzezińska-Błaszczyk E (2015). Cathelicidin impact on inflammatory cells. Cent Eur J Immunol.

[18] Lavigne R, Briers Y, Hertveldt K, Robben J, Volckaert G (2004). Identification and characterization of a highly thermostable bacteriophage lysozyme. Cell Mol Life Sci.

[19] Plotka M (2014). Novel highly thermostable endolysin from Thermus scotoductus MAT2119 bacteriophage Ph2119 with amino acid sequence similarity to eukaryotic peptidoglycan recognition proteins. Appl Environ Microbiol.

[20] Briers Y, Lavigne R (2015). Breaking barriers: expansion of the use of endolysins as novel antibacterials against Gram-negative bacteria. Future Microbiol.

[21] Shen MY, Sali A (2006). Statistical potential for assessment and prediction of protein structures. Protein Sci.

[22] Webb B, Sali A (2017). Protein Structure Modeling with MODELLER. Methods Mol Biol.

[23] Baldwin AJ, Inoue K (2006). The most C-terminal tri-glycine segment within the polyglycine stretch of the pea Toc75 transit peptide plays a critical role for targeting the protein to the chloroplast outer envelope membrane. FEBS J.

[24] Michel T, Reichhart JM, Hoffmann JA, Royet J (2001). *Drosophila* Toll is activated by Gram-positive bacteria through a circulating peptidoglycan recognition protein. Nature.

[25] Kumar S (2005). Selective recognition of synthetic lysine and meso-diaminopimelic acid-type peptidoglycan fragments by human peptidoglycan recognition proteins I{alpha} and S. J Biol Chem.

[26] Chen W (2013). Structural and Energetic Basis of Carbohydrate–Aromatic Packing Interactions in Proteins. Journal of the American Chemical Society.

[27] Sandalova T (2016). The crystal structure of the major pneumococcal autolysin LytA in complex with a large peptidoglycan fragment reveals the pivotal role of glycans for lytic activity. Mol Microbiol.

[28] Meroueh SO (2006). Three-dimensional structure of the bacterial cell wall peptidoglycan. Proc Natl Acad Sci USA.

[29] Razvi A, Scholtz JM (2006). Lessons in stability from thermophilic proteins. Protein Sci.

[30] Pucci F, Rooman M (2017). Physical and molecular bases of protein thermal stability and cold adaptation. Curr Opin Struct Biol.

[31] Kozlowski LP (2017). Proteome-pI: proteome isoelectric point database. Nucleic Acids Res.

[32] Dougherty DA (1996). Cation-pi interactions in chemistry and biology: a new view of benzene, Phe, Tyr, and Trp. Science.

[33] Pack SP, Yoo YJ (2004). Protein thermostability: structure-based difference of amino acid between thermophilic and mesophilic proteins. J Biotechnol.

[34] Morgan AA, Rubenstein E (2013). Proline: the distribution, frequency, positioning, and common functional roles of proline and polyproline sequences in the human proteome. PLoS One.

[35] Panja AS, Bandopadhyay B, Maiti S (2015). Protein Thermostability Is Owing to Their Preferences to Non-Polar Smaller Volume Amino Acids, Variations in Residual Physico-Chemical Properties and More Salt-Bridges. PLoS One.

[36] Sokalingam S, Raghunathan G, Soundrarajan N, Lee SG (2012). A study on the effect of surface lysine to arginine mutagenesis on protein stability and structure using green fluorescent protein. PLoS One.

[37] Kaczorowski T, Szybalski W (1996). Co-operativity of hexamer ligation. Gene.

[38] Stefanska A (2014). Discovery and characterization of RecA protein of thermophilic bacterium *Thermus thermophilus* MAT72 phage Tt72 that increases specificity of a PCR-based DNA amplification. J Biotechnol.

[39] Stefanska A (2016). Highly thermostable RadA protein from the archaeon *Pyrococcus woesei* enhances specificity of simplex and multiplex PCR assays. J Appl Genet.

[40] Bradford MM (1976). A rapid and sensitive method for the quantitation of microgram quantities of protein utilizing the principle of protein-dye binding. Anal Biochem.

[41] Adams PD (2010). PHENIX: a comprehensive Python-based system for macromolecular structure solution. Acta Crystallogr D Biol Crystallogr.

[42] Emsley P, Lohkamp B, Scott WG, Cowtan K (2010). Features and development of Coot. Acta Crystallogr D Biol Crystallogr.

[43] Desmarais, S. M., Cava, F., de Pedro, M. A. & Huang, K. C. Isolation and preparation of bacterial cell walls for compositional analysis by ultra performance liquid chromatography. *J Vis Exp*, e51183, 10.3791/51183 (2014). 10.3791/51183 PMC3987682 24457605

[44] Schuck P (2000). Size-distribution analysis of macromolecules by sedimentation velocity ultracentrifugation and lamm equation modeling. Biophys J.

