# Supplementary material for: Structure and function of the Ts2631 endolysin of Thermus scotoductus phage vB_Tsc2631 with unique N-terminal extension used for peptidoglycan binding
Source: Sci Rep. 2019 Feb 4;9:1261. doi: 10.1038/s41598-018-37417-6 (PMC6361986; doi:10.1038/s41598-018-37417-6)
Supplement: Supplementary file 1 — Supplementary Information [file 41598_2018_37417_MOESM1_ESM.pdf]

Structure and function of the Ts2631 endolysin of *Thermus scotoductus* phage vB_Tsc2631 with unique N-terminal extension used for peptidoglycan binding

Magdalena Plotka[1*], Enea Sancho-Vaello[2], Sebastian Dorawa[1], Anna-Karina Kaczorowska[3], Lukasz P. Kozlowski[4], Tadeusz Kaczorowski[1*], Kornelius Zeth[5]

[1]Laboratory of Extremophiles Biology, Department of Microbiology, Faculty of Biology, University of Gdansk, Gdansk, Poland

[2]Unidad de Biofisica, Centro Mixto Consejo Superior de Investigaciones Científicas-Universidad del País Vasco/Euskal Herriko Unibertsitatea (CSIC,UPV/EHU), Leioa, Bizkaia, Spain

[3]Collection of Plasmids and Microorganisms, University of Gdansk, Gdansk, Poland

[4]Institute of Informatics, Faculty of Mathematics, Informatics and Mechanics, University of Warsaw, Warsaw, Poland

[5]Department of Science and Environment, Roskilde University, Roskilde, Denmark

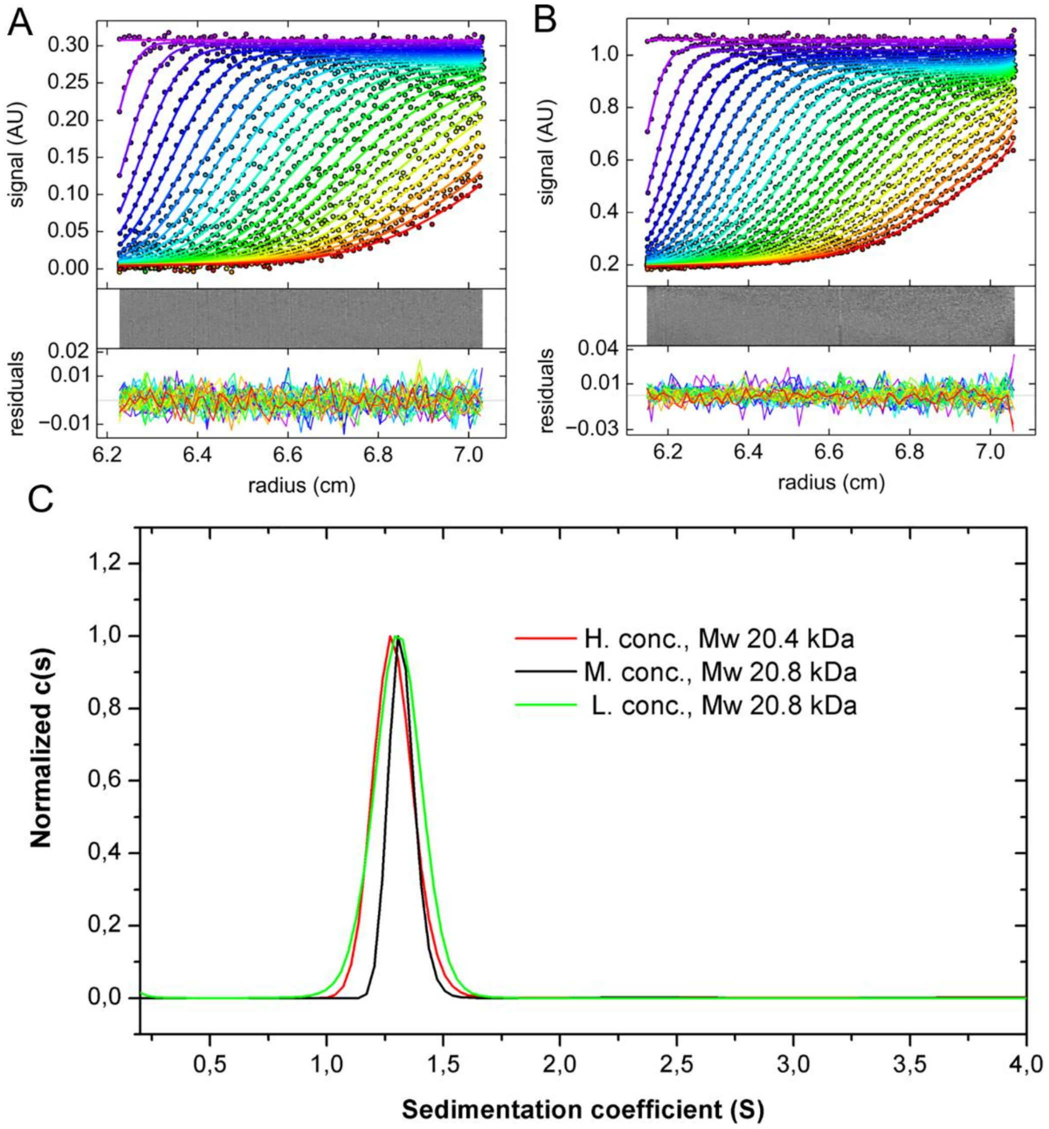

Supplementary Fig. S1 Determination of Ts2631 endolysin subunit composition by analytical ultracentrifugation. Its sedimentation velocity determined that Ts2631 endolysin is present in solution in the monomeric form. Sedimentation-velocity data (dots; color representing times in the order purple-blue-green-yellow-red) were collected for Ts2631 endolysin samples with (A) 0.34 OD and (B) 1.1 OD at 280 nm and are overlaid with the best-fit curves (lines), which were obtained from sedimentation coefficient distribution analysis. For clarity, only every third scan and every third data point are included. (C) Residuals of the experimental fits. Sedimentation coefficient distribution ([c(s)]) for three samples of Ts2631 endolysin (0.34 OD_{280} – green, 0.62 OD_{280} – black and 1.10 OD_{280} – red). At all concentrations, Ts2631 endolysin exhibits only a monomer peak.

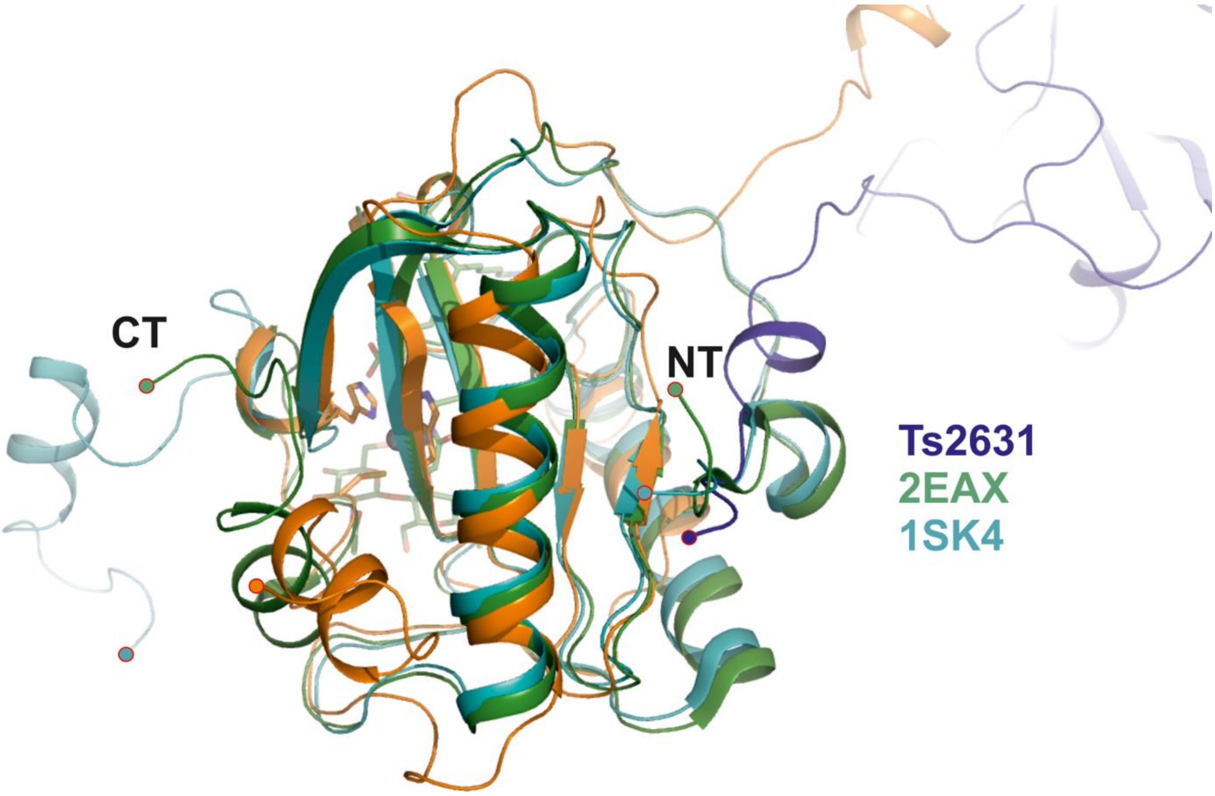

Supplementary Fig. S2 Structural similarity of Ts2631 endolysin and mammalian PGRPs. Superposition of human PGRP-Iα[1] and PGRP- IβC[2] (PDB entries: 1SK4 and 2EAX, respectively) onto Ts2631 endolysin.

```
                                                    APP
ENDO       1   MRI---LEPWNRWY----RQKRAYRVR--LTPIHYVVLHHTA--GP--EN-------QTPEAIKRYHEE-   49
1LBA       1   ------------------------AKQ--RESTDAIFVHCSA--TKPSQN-------VGVREIRQWHKE-   34
2Y28       1   MLLDEGWLAEARRV-----PSPHYDCRPDDENPSLLVVHNISLPPG-EFG---------GPWIDALFTGT   55
2EAX       1   -------CPGIVPRSVWGARETHCPRM--TLPAKYGIIIHTA--GR--TCNISDECRLLVRDIQSFYID-   56
PGLYRP2  375   ---AFLGCPAIHPRCRWGAAPYRGRPKLLQLPLGFLYVHHTYVPAPPCTDFTRCAANMRSMQRYHQDTQ-  440

                               P   A             P      P             P
ENDO      50   -------------ARGWPHIGYHYLVYRDGRVYKTLPNNAVPICCVR-------EFNPVSICCVAAVGDF   99
1LBA      35   --------------QGWLDVGYHFIIKRDGTVEAGRDEMAVGSHAK--------GYNHNSI-GVCLVGGI   81
2Y28      56   IIDPNAHPYFAGIAHLR--VSAHCLIRRDGEIVQYVPFDKRAWHAGVSSYQGRERCNDFSI-GIELEGT-  121
2EAX      57   -------------RLKSCDIGYNFLVGQDGAIYEGVGWNVQGSSTP--------GYDDIAL-GITFMGTF  104
PGLYRP2  441   ---------------GWGDIGYSFVVGSDGYVYEGRGWHWVGAHTL--------GHNSRGF-GVAIVGNY  486

                                                         A   P   PPA
ENDO     100   -----SAGVWPDDAPGWRALWELKQALAK--AY-PKA--LFVLHKNLVP--TECPG-RLTWELIQ--RKG  154
1LBA      82   DDKGKFDANFT--PAQMQSLRSLLVTLLA--KY-EGA--VLRAHHEVAP--KACPS-FDLKRWWEK-NEL  140
2Y28     122   -----DTLAYT--DAQYQQLAAVTNALIT--RY-PAIANNMTGHCNIAPERKTDPGPSFDWARFR--A--  177
2EAX     105   -----TGIPPN--AAALEAAQDLIQCAMVKGYLTPNY--LLVGHSDVAR--TLSPGQAL-YNIISTWP--  160
PGLYRP2  487   -----TAALPT---EAALRTVRDTLPSCAVRAGLLRPDYALLGHRQLVR--TDCPG-DALFDLLRTWP--  543

ENDO     155   GGGQ----   158
1LBA     141   --VTSDRG   146
2Y28     178   -LVTP---   181
2EAX     161   --HFKH--   164
PGLYRP2  544   --HFTA--   547
```

Supplementary Fig. S3 Structural sequence alignment of Ts2631 endolysin, T7L, AmpD, PGRP-Iβ and PGLYRP2. Structural alignment of the five PGN-binding proteins with important residues highlighted and marked: **A** cyan – active-site residues holding the zinc ion; **P** navy blue – PGN-binding sites according to National Center for Biotechnology Information protein annotation resource https://www.ncbi.nlm.nih.gov/protein/677570412[3]. Yellow shading indicates 100% identity. PDB entry for T7L is 1LBA, for AmpD is 2Y28 and for PGRP- Iβ is 2EAX; GenBank accession number PGLYRP2 – AF384856.

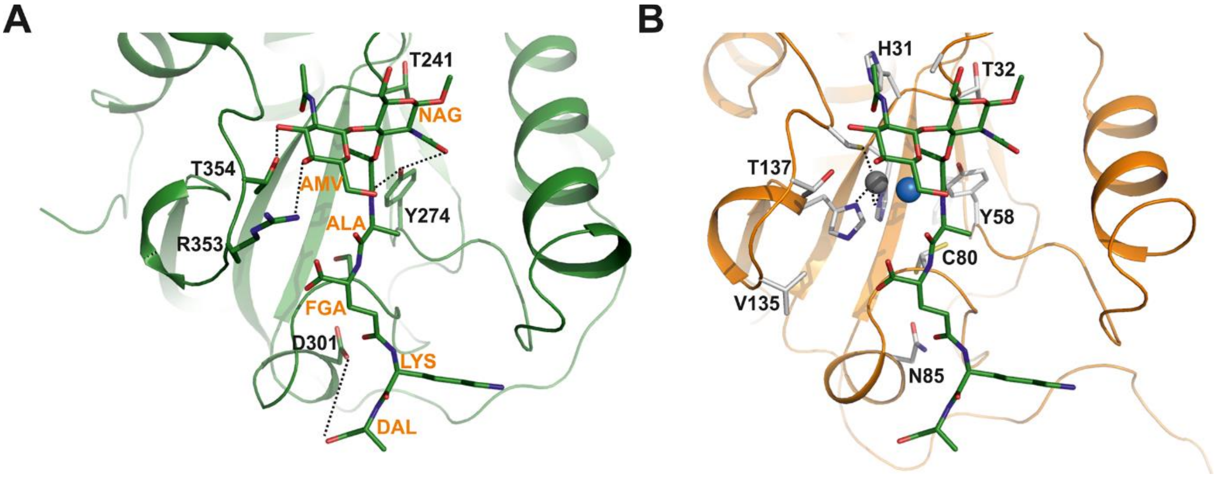

Supplementary Fig. S4 Residues involved in peptidoglycan binding. (A) Structure of human PGRP-Iβ (PDB entry 2EAX) shown in ribbon representation containing the PGRP ligand (NAG-NAM-l-Ala-γ-d-Glu-l-Lys-d-Ala), which is highlighted in stick representation to indicate residues interacting with a glucosamyl muramyl tetrapeptide[2]. (B) Structure of Ts2631 endolysin with highlighted residues in the PGN-binding groove.

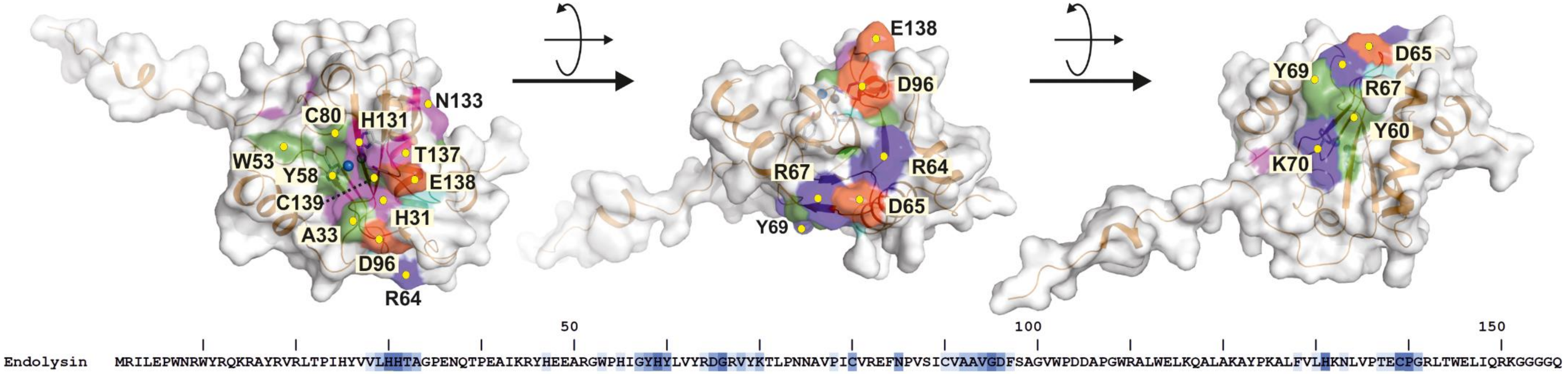

Supplementary Fig. S5 Surface representation of Ts2613 endolysin with conserved residues highlighted. Based on a multiple sequence alignment, conserved residues on the surface of Ts2631 endolysin were identified. These residues cluster around the active site (left picture) and on the opposite side of the molecule[4].

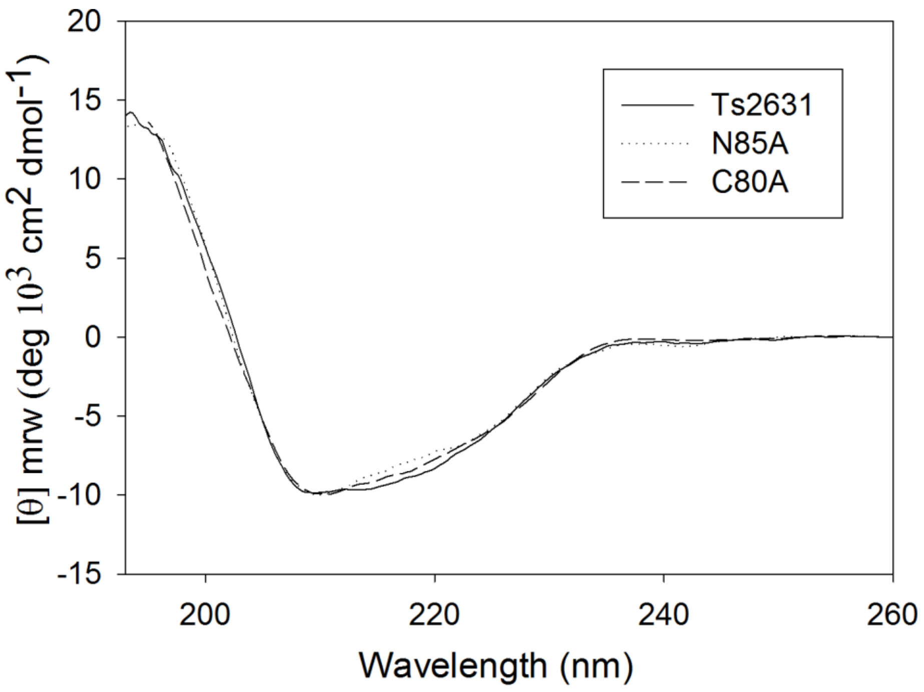

Supplementary Fig. S6 Far-UV circular dichroism spectra of wild-type Ts2631 endolysin and its C80A and N85A substitution variants. The spectra were convergent, suggesting that mutagenesis did not influence the folded structure. Proteins were dissolved in 10 mm potassium phosphate buffer, pH 8.0, with 150 mM ammonium sulfate. Spectra were taken at protein concentrations of 0.15 mM at room temperature. Curves represent an average of six scans, and blank buffer spectra were subtracted to compensate for solvent contributions.

Unique N-terminal extension
rich in Arg residues

```
Ts2631            MRILEPWNRWYRQKRAYRV-RLTPIHYVVLHHTAG-PEN--Q--------TPEA-IKRYH-E----EARGWPHIGYHYLVY-RDGRVYKTLPN--N-AVPICVREFNPVSICVAAVGD-FS-----
Ph2119            MRILEPWNRWYRQKGVYRI-RGTPPHYIVLHHTAG-PVD--Q--------APEV-IRDFH-E----KGRGWPHIGYHYLVY-QDGRVYKTLPN--N-AIPICVREFNPVSLCIAAVGD-FS-----

AKG94538.1__22-   ----------------------TPVTRIVLHYSAT-YPD--Q------DIGAAE-IRQMH------RARGWNDIGYHYVIR-RDGRVEPGRAE--N-VVGAHVAGHNTGSIGICCVGG-IERATGP
ALT58472.1__43-   -------------------------------------------------------IRQWH-K-----EQGWLDVGYHFIIR-RDGTVEAGRDQ--D-AVGSHVKGYNSTSVGVCLVGG-ID-----
YP_009291547.1_   --------------------------YIFVHCSAT-RPS--Q------DVGVRE-IRQWH-K-----ERGWLDVGYHYVIK-RDGTIETGRSL--E-AVGSHVKGYNEVSVGICLVGG-ID-----
YP_002003804.1_   -------------------------------------------------------IRQWH-K-----EQGWLDVGYHFIIR-RDGTVEAGRDQ--D-AVGSHVKGYNSTSVGVCLVGG-ID-----
AAX62800.1__43-   -------------------------------------------------------IRQWH-K-----EQGWLDVGYHFIIR-RDGTVEAGRDQ--D-AVGSHVKGYNSTSVGVCLVGG-ID-----
YP_002048642.1_   --------------------------YIFVHCSAT-RPS--Q------DVGVRE-IRQWH-K-----EQGWLDVGYHFVIK-RDGTIETGRSL--E-AVGSHVKGYNEVSVGICLVGG-ID-----
YP_003347533.1_   -------------------------------------------------------IRQWH-K-----EQGWLDVGYHFIIR-RDGTVEAGRDQ--D-AVGSHVKGYNSTSVGVCLVGG-ID-----
YP_004678738.1_   ----------------------------FVHCSAT-KAT--M------NIGVRE-IRQWH-K-----EQGWLDVGYHFIIR-RDGTVEVGRDQ--D-AVGSHVKGYNSTSVGVCLVGG-ID-----
YP_006990217.1_   ----------------------------FVHCSAT-KAT--Q------DVGVRE-IRQWH-K-----EQGWLDVGYHFIIR-RDGTVEDGRDV--N-AVGSHARGNNHNSVGVCLVGG-ID-----
YP_009324956.1_   ----------------------------------------------------ET-IRMWH-K-----QQGWLDVGYHFIIK-RDGTVEEGRPV--N-VVGSHVKDWNSRSVGVCLVGG-ID-----
YP_009190970.1_   -------------------------------------------------------IRQWH-K-----EQGWLDVGYHFIIR-RDGTVEAGRDQ--D-AVGSHVKGYNSTSVGVCLVGG-ID-----
YP_007517774.1_   --------------------------YIVIHCSQT-RPS--Q------KIGAKD-IDRWH-R-----ERGWLKIGYAKVIK-RDGTVEQGRADDEL-Q--AHVKEYNHVSTSVCVVGGAV-----E
YP_009200607.1_   -----------------------VTEAIFVHCSAT-KAT--M------NVGLRE-IRQWH-K-----EQGWLDVGYHFIIR-RDGTIEEGRPV--D-VVGSHVKDWNSKSVGVCLVGG-ID-----
YP_009292485.1_   ----------------------------------------------------ET-IRMWH-K-----QQGWLDVGYHFIIK-RDGTVEEGRPV--N-VVGSHVKDWNSRSVGVCLVGG-IN-----
YP_009324495.1_   ----------------------------------------------------ET-IRMWH-K-----QQGWLDVGYHFIIK-RDGTVEEGRPV--N-VVGSHVKDWNSRSVGVCLVGG-ID-----
YP_002003756.1_   ----------------------------------------------------ET-IRMWH-K-----QQGWLDVGYHFIIK-RDGTVEEGRPV--N-VVGSHVKDWNSKSVGVCLVGG-ID-----
CAJ29362.1__41-   ----------------------------------------------------ET-IRMWH-K-----QQGWLDVGYHFIIK-RDGTVEEGRPV--N-VVGSHVKDWNSRSVGVCLVGG-IN-----
YP_009044268.1_   ----------------------------------------------------ET-IRMWH-K-----QQGWLDVGYHFVIK-RDGTVEEGRPV--N-VVGSHVKDWNSRSVGVCLVGG-IN-----
BAR32142.1__26-   --------------------------YIVVHCSQT-RPS--Q------KIGAKE-IDRWH-R-----ERGWLKIGYARVIK-RDGTVEQGR--DDD-ELQAHVKNYNHISTSVCVVGG-AKE---E
AOZ65543.1__43-   -------------------------------------------------------ITQWH-K-----EQGWLAIGYHFVIR-RDGTVEAGRDQ--D-AVGSHVKGYNSTSVGVCLVGG-ID-----
AOZ65493.1__43-   -------------------------------------------------------IAQWH-K-----EQGWLAIGYHFVIR-RDGTVEAGRDQ--D-AVGSHVKGYNSTSVGVCLIGG-ID-----
YP_009303665.1_   -----------------------VTEAIFVHCSAT-KAT--M------NVGLRE-IRQWH-K-----EQGWLDVGYHFIIR-RDGTIEEGRPV--D-VVGSHVKDWNSKSVGVCLVGG-ID-----
YP_009204343.1_   -----------------------VTEAIFVHCSAT-KAT--M------NVGLRE-IRQWH-K-----EQGWLDVGYHFIIR-RDGTIEEGRPV--G-VVGSHVKDWNSKSVGVCLVGG-ID-----
YP_009007152.1_   ----------------------------------------------------ET-IRMWH-K-----QQGWLDVGYHFIIK-RDGTVEEGRPV--N-VVGSHVKDWNSRSVGVCLVGG-ID-----
YP_008241819.1_   ---------------------MRTIKYIVLHCTAT-VQH--A--------KVENIIKYWR-N-----VLKWKSNGYHFIIE-SSGKVHNITPI--E-NVANGVAGYNSASIHISYIGG-ID-----
YP_009285908.1_   ---------------------MRPIREIVIHCSAT-PP--DWKSGDSLFEKRAE-IRRWH-V----QGNGWSDIGYHYLID-RDGHCAMGRP---LARIGAHVRGHNTGTIGLCLIGG-HGAS-AD
YP_004300550.1_   -----------------------MTEAIFVHCSAT-KAS--M------NVGLRE-IRQWH-K-----EQGWLDVGYHFIIR-RDGTIEEGRPV--D-VVGSHVKDWNSKSVGVCLVGG-ID-----
YP_006383592.1_   ---------------------------IIVHHSEH-STA-----------NVYD-IDRWH-K-----DKGWCGIGYHYFID-KQGNIYTGRPE--D-WTGAHCIDHNTKSIGICLQGR-LQ-----
YP_009152476.1_   -------------------------TDAIFVHCSAT-KSS--Q------NVGVRE-IRQWH-K-----EQGWLDVGYHYIIK-RDGTVEEGRDE--M-AVGSHAKGHNHNSIGVCLVGG-ID-----
YP_009187285.1_   K-------------VQFTP--RKETNAIFVHCAAT-KPS--Q------DVGVRE-IRQWH-K-----EKGWLDIGYHFVIR-RDGTVEAGREQ--S-AVGSHVANYNHDSVGVCLVGG-ID-----
AKH48662.1__21-   ---------------------MRNINEIIIHCSAT-REG--Q------DISVDT-IREWH-V----DGRGWSDIGYHFYIE-LDGSIKKGRDI--D-KTGAHCKGHNRNSVGICYCGG-VE-----
YP_008766731.1_   -------------------------------------------------------ISQWH-R-----EQGWLAIGYHFVIR-RDGTIEEGRPV--D-VVGSHVKDWNSKSVGVCLVGG-ID-----
YP_009279747.1_   -----------------------VTDAIFVHCSAT-QPS--Q------DIGVDT-IRMWH-K-----QQGWLDVGYHFVIK-RDGTVEEGRPV--D-VVGSHVKDWNYRSVGVCLVGG-ID-----
YP_009291491.1_   -------------------------TDAIFVHCSAT-KPS--Q------NVGVRE-IRQWH-K-----EQGWLDVGYHFIIK-RDGTVEEGRDE--M-AVGSHAKGYNHNSIGVCLVGG-ID-----
YP_002308397.1_   -----------------------VTEAIFVHCSAT-KAT--M------NVGLRE-IRQWH-K-----EQGWLDVGYHFIIR-RDGTIEEGRPV--D-VVGSHVKDWNSKSVGVCLVGG-ID-----
YP_009004176.1_   -------------------------------------------------------IEMWH-K-----QQGWLAVGYHFIIK-RDGTVEEGRPV--D-VVGSHVKDWNHKSVGVCLVGG-ID-----
ANN86257.1__22-   -------------------------TEAIFVHCSAT-KQS--Q------NIGVRE-IRQWH-K-----EQGWLDVGYHFIIK-RDGTVEAGRDE--L-AVGSHVKGYNHNSVGVCLVGG-ID-----
AAP34083.1__21-   -----------------------FTDAIFVHCSAT-KPS--Q------NVGVRE-IRQWH-K-----EQGWLDVGYHFIIK-RDGTVEAGRDE--M-AVGSHAKGYNHNSIGVCLVGG-ID-----
BAR33340.1__26-   --------------------------YIVIHCSQT-RPS--Q------DVGASE-IDQWH-R-----QRGWLKIGYGKVIK-RDGTVEQGR--GDN-EVQAHVKGYNHNSYGLCLVGG-AK-----
YP_338105.1__41   ----------------------------------------------------ET-IRMWH-K-----QQAWLDVGYHFIIK-RDGTVEEGRPV--N-VVGSHVKDWNSRSVGVCLVGG-IN-----
NP_848277.1__43   -------------------------------------------------------IRQSH-K-----ERGFLDVGYHFIIK-RDGTVEEGRDE--M-AVGSHVKGYNHNSIGVCLVGG-ID-----
ANS05754.1__26-   --------------------------YIVIHCSQT-RPS--Q------KIGAKE-IDRWH-R-----EKGWLKIGYGTVIK-RDGTIEQGRGDDD---VQAAVKGYNHTSFNLCLIGG-AK----E
YP_009196355.1_   -------------------------------------------------------IRQWH-K-----EQGWLDIGYHFVIR-RDGTIETGRDL--G-AVGSHVKGYNETSVGVCLVGG-ID-----
CAJ29363.1__49-   -------------------------------------------------------------K-----QQGWLDVGYHFIIK-RDGTVEEGRPV--N-VVGSHVKDWNSRSVGVCLVGG-IN-----
NP_052084.1__22   -------------------------TEAIFVHCSAT-KPS--Q------NIGVRE-IRQWH-K-----EQGWLDVGYHFIIK-RDGTVEAGRDE--L-AVGSHVKGYNHNSVGVCLVGG-ID-----
YP_009286644.1_   -------------------------TEAIFVHCSAT-KPS--Q------NIGVRE-IRQWH-K-----EQGWLDVGYHFIIK-RDGTVEAGRDE--L-AVGSHVKGYNHNSVGVCLVGG-ID-----
YP_009187966.1_   ----------------------TATDYLVVHCAAT-KAS--M------DIGVRE-IRQWH-V-----QKGWLDIGYHFVIR-RNGTVEDGRPH--N-VIGAHVEGFNSRSLGICLAGG-ID-----
YP_009198323.1_   -------------------------TEAIFVHCSAT-KPS--Q------NIGVRE-IRQWH-K-----EQGWLDVGYHFIIK-RDGTVEAGRDE--L-AVGSHVKGYNHNSVGVCLVGG-ID-----
AKQ06778.1__22-   -------------------------TEAIFVHCSAT-KPS--Q------NIGVRE-IRQWH-K-----EQGWLDVGYHFIIK-RDGTVEAGRDE--L-AVGSHVKGYNHNSVGVCLVGG-ID-----
```

```
YP_009205667.1_   ------------------------TEAIFVHCSAT-KPS--Q------NIGVRE-IRQWH-K-----EQGWLDVGYHFIIK-RDGTVEAGRDE--L-AVGSHVKGYNHNSVGVCLVGG-ID-----
YP_004306666.1_   R-------------VQFKP--RESTEAIFVHCSAT-KPN--Q------NVGVRE-IRQWH-K-----EQGWLDVGYHFIIK-RDGTVEAGRDE--M-AVGSHAKGYNHNSIGVCLVGG-ID-----
YP_009206322.1_   RSK-----------GSYRDFGAASKTHIIVHHSLT-KTG-----------SAEA-YARYH-V----DTLGWPGIGYHFVIE-QDGTIKQNHSI--G-VMSYHVGNHNKYCVGICLTGD-FR-----
YP_002003950.1_   ------------------------TDAIFVHCSAT-KPS--Q------NVGVRE-IRQWH-K-----ERGFLDVGYHFIIK-RDGTVDAGRDE--M-AVGSHAKGYNHNSIGVCLVGG-ID-----
YP_918995.1__43   -------------------------------------------------------ISQWH-K-----EQGWLAIGYHFVIR-RDGTIEEGRPV--E-VIGSHVKSWNSKSVGVCLVGG-ID-----
YP_002003466.1_   ----------------------------FVHCSAT-KAT--M------NVGLRE-IRQWH-K-----EQVWLDVGYHFIIS-RDGRIEEGRPV--G-VVGSHVKDWNSKSVGVCLVGG-ID-----
NP_523313.1__22   ------------------------TEAIFVHCSAT-KPS--Q------NVGVRE-IRQWH-K-----EQGWLDVGYHFIIK-RDGTVEAGRDE--L-AVGSHAKGYNHNSIGVCLVGG-ID-----
CAA24346.1__22-   ------------------------TDAIFVHCSAT-KPS--Q------NVGVRE-IRQWH-K-----EQGWLDVGYHFIIK-RDGTVEAGRDE--M-AVGSHAKGYNHNSIGVCLVGG-ID-----
AAP33982.1__22-   ------------------------TDAIFVHCSAT-KPS--Q------NVGVRE-IRQWH-K-----EQGWLDVGYHFIIK-RDGTVEAGRDE--M-VVGSHAKGYNHNSIGVCLVGG-ID-----
BAR33010.1__22-   ----------------------TKTDMIVIHCSAT-PAD--M------DVDAAK-IKHWH-T----VDNGWDDIGYHYVIK-RDGTLETGREE--H-RTGSHARQVNGTSIGICMIGG-SD-----
YP_009014835.1_   -------------------------------------------------------ISQWH-K-----EQGWLAIGYHFVIR-RDGTIEEGRPV--E-VIGSHVKSWNSKSVGVCLVGG-ID-----
YP_009100091.1_   ---------------------MRPIDTLVWHCAAT-PE--GK------WFDVDD-VRKWH-TLPPPKGRGWKDVGYHFIIL-LDGTIQKGRPL--D-RVGAHVEGHNTGSIGCCYIGG-VDAITL-
APD19632.1__43-   -------------------------------------------------------ISQWH-K-----ELGWLAIGYHFVIR-RDGTIEEGRPV--E-VVGSHVKDWNSKSVGVCLVGG-IN-----
YP_001949762.1_   ------------------------TEAIFVHCSAT-KPS--Q------NIGVRE-IRQWH-K-----EQGWLDVGYHFIIK-RDGTVEAGRDE--L-AVGSHVKGYNHNSVGVCLVGG-ID-----
YP_006560261.1_   ---------------------------IVIHCSDT-PNG--R------DDTAED-IHGWH-L-----AKGWDGIGYHHVIE-VGGAVRAGR--PHY-WIGSHVAGYNEDSLGICMIGR-D-----Q
YP_009100934.1_   ---------------------MRPIRKIVIHCADT-FH--DM------GVGADW-VRNIH-V----NENGWRDIGYHYVIT-RDGEVEKGRDE--A-IAGAHVRGHNANSIGVCLIGG-KARA---
YP_001552293.1_   ---------------------------IIIHCAAT-PN--GKH------FTAKD-IHDWH-L----E-RGWSGIGYHYVIR-VDGVLEVGRPE--YW-TGAHAKGHNTGSIAICLIGT--------
CUR50697.1__22-   ----------------------TATDYLVVHCAAT-KAS--M------DIGVRE-IRQWH-V-----QKGWLDIGYHFVIR-RNGTVEDGRPH--N-VIGAHVEGFNARSLGICLAGG-ID-----
ADX87661.1__16-   ---------------AFKK--RTQTKAILVHCTAT-KPH--Q------DIDVRE-VRQWHTR-----DNGWFDVGYHFIIR-RNGVIENGRPV--D-VVGAHASGFNSDSVGVCLVGG-IN-----
AEI71112.1__23-   -----------------------PVDEIIVHCSATRPDWMANAP---FADQVAE-IRRWH-M----QDRGWRNIGYHWLIS-RSGQVLAGRPE--T-EIGAHVVDHNRGTIGICLIGG-AGSAETD
AGC35529.1__26-   --------------------------YLVVHCSAT-QPK--M------DIGAKE-IRQWH-R-----EKGWIDIGYHFVIR-RDGTVELGRPE--N-VVGAHVENHNSNSIGICLVGG-VD-----
YP_009216701.1_   --------------------------YIIYHHAEH-G-N--C------S--VFD-IHNWH-L-----NNSWSGIGYNFFVR-KDGSIWEGR--PID-AMGAHAVGYNDISIGICAEGK-Y-----A
BAQ94242.1__26-   --------------------------YIVIHCSQT-RPS--Q------KWGAKE-IDRVH-R-----EFGWTKIGYGKVIK-RDGTVEQGR--EDD-AVQAHVKGYNHTAYGLCLIGG-AKE---E
YP_009191815.1_   K-------------TPFKK--RLKTNTIFIHAADT-PST--M------DIGWKE-IHQWH-V----KDNGWAAIGYHVVIR-RDGTIEAGR--PLD-AVGAHVASRNSDSVGICLIGG-K-----G
YP_003347926.1_   ---------------AFKK--RTQTKAILVHCTAT-KPH--Q------DIGVRE-VRQWHTR-----DNGWFDVGYHFIIR-RNGVIENGRPV--D-VVGAHASGFNSDSVGVCLVGG-IN-----
YP_007005440.1_   ---------------------------LIVHCAAT-PPS--M------NIGLRE-IRQWH-K-----EKGWLDVGYHFIIR-RDGTIEDGRDV--G-AVGAHCEGKNYTSVGVCLVGG-VD-----
YP_249586.1__27   ---------------------------ILVHCTAT-KPH--Q------DIGVRE-VRQWHTR-----DNGWFDVGYHFIIR-RNGVIENGRPV--D-VVGAHASGFNSDSIGVCLVGG-IN-----
ADX87518.1__16-   ---------------AFKK--RTQTKAILVHCTAT-KPH--Q------DIGVRE-VRQWHTR-----DNGWFDVGYHFIIR-RNGVIENGRPV--D-VVGAHASGFNSDSVGVCLVGG-IN-----
YP_004306332.1_   ----------------------TSTNLIVVHCAAT-KPT--M------DVGRKE-IQMWH-V-----QQGWLAIGYHFVIR-RDGTIEEGRPH--D-VVGSHVKSRNADSVGICLVGG-ID-----
YP_009217953.1_   --------------------------YIIYHHAEH-G-N--C------S--VYD-IHNWH-L----NNSWSGIGYNFFVR-KDGSIWEGR---PID-AMGAHAVGYNDIAVGICAEGK-Y-----A
BAR16484.1__26-   --------------------------YVVIHCADT-PAD--M------DVGAAD-IRRWH-V----DERGWDDVGYHWIVK-RSGQLEPGRDQ--R-LQGSHALAVNSKSIGVCLVGR--------
YP_006488655.1_   ---------------------------IIVHHSEH-STA-----------NVYD-IDKWH-K-----DKGWCGIGYHYFID-KQGNIYTGRPE--D-WTGAHCINHNTKSIGICLQGR-LQ-----
BAQ86927.1__26-   --------------------------YVVIHCADT-PAD--M------DVGAAD-IRRWH-V----DERGWDDVGYHWIIK-RSGQLEPGRDQ--R-LQGSHALAVNSKSIGVCLVGR--------
YP_004286199.1_   ----------------FKE--RLSTKMIVVHCSAT-KAS--M------DIGRKE-IQMWH-V-----QQGWLAIGYHLVIR-RDGTIEQGRPH--K-AIGSHVKGHNSDSIGICLVGG-ID-----
BAQ86653.1__26-   --------------------------YVVIHCADT-PAD--M------DVGAAD-IRRWH-V----DERGWDDVGYHWIIK-RSGQLEPGRDQ--R-LQGSHALAVNSKSIGVCLVGR--------
ANS03019.1__21-   ---------------------MRKINKIIIHCSAT-PEG--R------EHDVAD-IRRWH-L-----KRGFNDIGYHYLIH-IDGTIEEGRPL--N-KQGAHCSTENKGSIGLCYVGG-MSK----
BAR33619.1__5-1   DE----WGAIDSGKRLSEF--RRVPVGIVVHHTTG-SAS-SP------WDRIRQ-HDKYHVK-----TRGWRSIAYNWLVSGETGEVFEGRGW--K--QGAATKGHNSNTTSISYIGS-GD-----

cons                                                                                    :   .*   :    *                     *
```

Unique C-terminal four-glycine stretch

```
Ts2631            A-----GV--W-----------PDDAPG-WRALWELKQ---------ALAKA--------YP-K-A--L-FV--LHKNL---V----P-T--ECPGRL---TWELIQRKGGGGQ
Ph2119            Q-----GP-AW-----------PDNAPG-WKALLELKD---------ALVKA--------YP-K-A--V-LV--LHKEL---T----Q-T--TCPGVL---SWGMVAEKGG---

AKG94538.1__22-   N---V--G--V--D--------NRTDAQ-RKATIALV--------R-DLLAR-------H--PR-AQV--L---GHRD--L-T-A----TQ--CPGFDVRTWWAQVNR-E----
ALT58472.1__43-   A---K--G-NP--E------A-NFTPQQ-MSALKGLL--------H-ELKG--T-----Y-P-K-A--V-IM--AHHD--V-A----P-K--ACP-------------------
YP_009291547.1_   D---Q--G-KP--N------A-NFTVAQ-MESLKHLL--------A-RTGE--T-----Y-P-Q-A--Q-IM--AHHD--V-A----P-K--ACP-------------------
YP_002003804.1_   A---K--G-NP--E------A-NFTPQQ-MSALNGLL--------H-ELRG--T-----Y-P-K-A--V-IM--AHHD--V-A----P-K--ACP-------------------
AAX62800.1__43-   A---K--G-NP--E------A-NFTPQQ-MSALNGVL--------H-ELRG--T-----Y-P-K-A--V-IM--AHHD--V-A----P-K--ACP-------------------
YP_002048642.1_   D---Q--G-KP--N------A-NFTVAQ-MESLKAVL--------K-EVGE--T-----Y-P-E-A--T-IM--AHHD--V-A----P-K--ACP-------------------
YP_003347533.1_   A---K--G-NP--E------A-NFTPAQ-MQALRSLL--------V-ELKV--Q-----Y-T-G-A--V-LM--AHHD--V-A----P-K--ACP-------------------
YP_004678738.1_   A---K--G-NP--E------A-NFTPAQ-MQALRSLL--------V-ELKV--Q-----Y-D-G-A--V-LM--AHHD--V-A----P-K--ACP-------------------
YP_006990217.1_   E---K--G-RH--E------A-NFTPQQ-MSALRTLL--------E-VLMD--K-----Y-P-S-A--V-LK--AHHD--V-A----P-K--ACP-------------------
YP_009324956.1_   A---K--G-KF--E------A-NFTPAQ-MDSLRSKL--------A-DLKA--L-----Y-P-Q-A--E-IK--AHHD--V-A----P-K--ACPS------------------
YP_009190970.1_   A---K--G-NP--E------A-NFTPAQ-MQSLRSLL--------V-ELKV--Q-----Y-A-G-A--V-LM--AHHD--V-A----P-K--ACP-------------------
YP_007517774.1_   E---N--W-KEPED--------NFTGEQ-WESLKKVL--------E-ELVIK-------FPE-G-A--V-LM--AHHD--V-A----P-K--ACP-------------------
```

```
YP_009200607.1_  D---K--G-KF--E------A-NFTPAQ-MQSLKEKL--------A-DLLD--M-----Y-P-A-R--I-V---GHYE--L-D----ERK--TCP-------------------
YP_009292485.1_  A---K--G-KF--E------A-NFTPAQ-MNSLRNKL--------A-DLKA--L-----Y-P-D-A--E-VK--AHHD--V-A----P-K--ACP-------------------
YP_009324495.1_  A---K--G-KF--E------A-NFTPAQ-MNSLRNKL--------A-DLKD--L-----Y-P-Q-A--E-IK--AHHD--V-A----P-K--ACPS------------------
YP_002003756.1_  A---K--G-KF--E------A-NFTPAQ-MNSLRNKL--------A-DLKA--M-----Y-P-Q-A--E-IK--AHHD--V-A----P-K--ACPS------------------
CAJ29362.1__41-  A---K--G-QF--E------A-NFTPAQ-MNSLRNKL--------D-DLKV--M-----Y-P-Q-A--E-IK--AHHD--V-A----P-K--ACPS------------------
YP_009044268.1_  A---N--G-KF--E------A-NFTPAQ-MDSLRIKL--------D-DLKV--L-----Y-P-Q-A--E-IR--AHHD--V-A----P-K--ACPS------------------
BAR32142.1__26-  D---W--Q-QP--E------D-NFTGEQ-WESLKKVL--------E-ELVIK-------YPE-Q-A--E-IK--AHHD--V-A----P-K--ACPS------------------
AOZ65543.1__43-  A---K--G-NP--E------A-NFTPAQ-MQSLRSLL--------V-ELKV--Q-----Y-A-A-R--I-V---GHYE--L-D----ERK--TCP-------------------
AOZ65493.1__43-  A---K--G-NP--E------A-NFTPAQ-MQSLRSLL--------V-ELKV--Q-----Y-A-G-A--V-LM--AHHD--V-A----P-K--ACP-------------------
YP_009303665.1_  D---K--G-RY--E------A-NFTPAQ-MHSLKEKL--------A-DLLD--M-----Y-P-D-A--E-VK--AHHD--V-A----P-K--ACP-------------------
YP_009204343.1_  D---K--G-KY--E------A-NFTPAQ-MHSLKEKL--------A-DLLD--M-----Y-P-D-A--E-VK--AHHD--V-A----P-K--ACP-------------------
YP_009007152.1_  A---N--G-KF--E------A-NFTPAQ-MNSLRNKL--------S-DLKV--L-----Y-P-Q-A--E-IK--AHHD--V-A----P-K--ACP-------------------
YP_008241819.1_  KN-----G-KA-LD--------NRTPEQ-IEAQKQLI--------R-TLTKQ-------F-P-N-A--E-IK--GHRDFPN-V----R-K--DCPS------------------
YP_009285908.1_  D---A-----F--E------D-HFTPAQ-DRALRALI--------A-ELQAR-------F---G-H-LE-VT--GHNL--Y-A-P----K--ACPG------------------
YP_004300550.1_  D---K--G-KH--E------A-NFTPAQ-MHSLKEKL--------A-DLLD--M-----Y-P-D-A--E-VK--AHHD--V-A----P-K--ACP-------------------
YP_006383592.1_  T------------E--------KVTDPQ-YKALLWLIQ---------DIKNR-R-------G-N-M--P-VY--GHKEL-N------S-T--DCPGNL---DLDKLRR------
YP_009152476.1_  D---K--G-KF--E------A-NFTPAQ-MQSLRSLL--------V-TLLA--K-----Y-E-G-S--V-LR--AHHD--V-A----P-K--ACP-------------------
YP_009187285.1_  D---K--G-KF--A------A-NFTPAQ-MQSLRSLL--------V-ELKV--Q-----Y-P-D-S--A-LR--AHHD--V-A----P-K--ACPS------------------
AKH48662.1__21-  S---D--G-KTPKD------T-R-TPIQ-KESLIHVLK---------TLKAM-------Y--PE-A-I--IY--SHNE--F-A-N----K--ACP-------------------
YP_008766731.1_  A---K--G-KF--E------A-NFTPAQ-MQSLKEKL--------A-DLKD--L-----Y-P-D-A--E-IK--AHHD--I-A----P-K--ACP-------------------
YP_009279747.1_  A---K--G-KF--E------A-NFTPAQ-MSALRNKL--------A-ELKA--L-----Y-P-Q-A--E-IK--AHHD--V-A----P-K--ACPS------------------
YP_009291491.1_  D---K--G-KF--D------A-NFTPAQ-MQSLRSLL--------V-TLLA--K-----Y-E-G-A--V-LK--AHHD--V-A----P-K--ACP-------------------
YP_002308397.1_  D---K--G-RH--E------A-NFTPAQ-MHSLKEKL--------A-DLKD--L-----Y-P-D-A--E-IK--AHHD--V-A----P-K--ACP-------------------
YP_009004176.1_  D---K--A-KF--A------A-NFTPAQ-MQSLKVKL--------D-ELKA--L-----Y-P-Q-A--V-IK--AHHD--V-A----P-K--ACP-------------------
ANN86257.1__22-  D---K--G-KF--D------A-NFTPAQ-MQALRSLL--------V-TLLA--K-----Y-E-G-S--I-LR--AHHD--V-A----P-K--ACP-------------------
AAP34083.1__21-  D---K--G-KF--D------A-NFTPAQ-MQSLRSLL--------V-TLLA--K-----Y-E-S-A--V-LR--AHHE--V-A----P-K--ACP-------------------
BAR33340.1__26-  EENWK--E-EE--D--------NYTAEQ-WESLKKTL--------E-ELLIK-------Y-P-D-A--Q-IV--GHRD--L-D----DSK--FCP-------------------
YP_338105.1__41  A---K--G-QF--E------A-NFTPAQ-MNSLRNKL--------D-DLKV--M-----Y-P-Q-A--E-IR--AHHD--V-A----P-K--ACPS------------------
NP_848277.1__43  D---K--G-KF--D------A-NFTPAQ-MQSLRSLL--------V-TLLA--K-----Y-E-G-A--V-LR--AHHD--V-A----P-K--ACP-------------------
ANS05754.1__26-  D---N--W-KE--E------EDNFTGEQ-WESLKTEL--------T-RLIKK-------Y-P-D-A--R-IV--GHRE--L-D----D-KK-FCP-------------------
YP_009196355.1_  A---K--G-NH--E------A-NFTPQQ-MASLKNVL--------T-TLKG--E-----W-P-N-A--V-IM--AHHD--V-A----P-K--ACP-------------------
CAJ29363.1__49-  A---K--G-QF--E------A-NFTPAQ-MNSLRNKL--------D-DLKV--M-----Y-P-Q-A--E-IR--AHHD--V-A----P-K--ACP-------------------
NP_052084.1__22  D---K--G-KF--D------A-NFTPAQ-MQALRSLL--------V-TLLA--K-----Y-E-G-S--I-LR--AHHD--V-A----P-K--ACP-------------------
YP_009286644.1_  D---K--G-KF--D------A-NFTPAQ-MQALRSLL--------V-TLLA--K-----Y-E-G-S--V-LR--AHHD--V-A----P-K--ACP-------------------
YP_009187966.1_  D---K--G-NP--Q------N-NFTPEQ-FNSLK-LL--------L-TAQK--R----TY-P-Q-A--K-IV--GHHD--LFA----G-K--ACPSFK---VSDWLQSVG----
YP_009198323.1_  D---K--G-KF--D------A-NFTPAQ-MQALRSLL--------V-TLLA--K-----Y-E-G-S--V-LR--AHHD--V-A----P-K--ACP-------------------
AKQ06778.1__22-  D---K--G-KF--D------A-NFTPAQ-MQALRSLL--------V-TLLA--K-----Y-E-G-S--V-LR--AHHD--V-A----P-K--ACP-------------------
YP_009205667.1_  D---K--G-KF--D------A-NFTPAQ-MQALRSLL--------V-TLLA--K-----Y-E-G-S--V-LR--AHHD--V-A----P-K--ACP-------------------
YP_004306666.1_  D---K--G-KF--D------A-NFTSSQ-MQSLRSLL--------V-TLLA--K-----Y-E-G-A--V-LR--AHHE--V-A----P-K--ACP-------------------
YP_009206322.1_  T-----QE---------------PTTAQ-KNSLCQLVS---------YLQRN--------YP-Q-L--KTVK--GHSDL---R-GYEW-K--ECP-------------------
YP_002003950.1_  D---K--G-KF--D------A-NFTPAQ-MQSLRSLL--------V-TLLA--K-----Y-E-G-A--V-LR--AHHD--V-A----P-K--ACP-------------------
YP_918995.1__43  D---K--G-HF--E------A-NFTPAQ-MVSLKEKL--------A-DLMN--M-----Y-P-E-A--E-VK--AHHD--V-A----P-K--ACP-------------------
YP_002003466.1_  D---K--G-RH--E------A-NFTPAQ-MHSLKEKL--------A-DLLD--M-----Y-P-D-A--E-VK--AHHD--V-A----P-K--ACP-------------------
NP_523313.1__22  D---K--G-KF--D------A-NFTPAQ-MQSLRSLL--------V-TLLA--K-----Y-E-G-S--V-LR--AHHD--V-A----P-K--ACP-------------------
CAA24346.1__22-  D---K--G-KF--D------A-NFTPAQ-MQSLRSLL--------V-TLLA--K-----Y-E-G-A--G-LR--AHHE--V-A----P-K--ACP-------------------
AAP33982.1__22-  D---K--G-KF--D------A-NFTPAQ-MQSLRSLL--------V-TLLA--K-----Y-E-G-A--V-LR--AHHE--V-A----P-K--ACP-------------------
BAR33010.1__22-  K---S--G-GW--E------N-NFLPEQ-FDTLKQIV--------E-TLKDK-------Y-SIT--KI--I---GHYE--V-D-D--V-KK--CP-------------------
YP_009014835.1_  D---K--G-HF--E------A-NFTPAQ-MVSLKEKL--------A-DLLD--M-----Y-P-D-A--E-VK--AHHD--V-A----P-K--ACP-------------------
YP_009100091.1_  ----K-----P-KD------T-R-TAAQ-KAAMLKLT--------Q-DLLKQ-------F-P---S-I--KRIAGHNE--Y-A-S----K--ACP-------------------
APD19632.1__43-  D---K--G-NH--E------A-NFTPAQ-MQSLKEKL--------A-DLLD--L-----Y-P-D-A--A-IK--AHHD--V-A----P-K--ACP-------------------
YP_001949762.1_  D---K--G-KF--D------A-NFTPAQ-MQALRSLL--------V-TLLA--K-----Y-E-G-S--V-LR--AHHD--V-A----S-K--ACP-------------------
YP_006560261.1_  FD-----G------------------DQ-WRALEGLV--------L-SLNAQ-------Y-P-D-A--E-VL--GHRD--L-D----SKK--TCP-------------------
YP_009100934.1_  ----------F--NGQHSNPA-NFTHKQ-WETLRNLV--------K-QLLLE-------Y-P-A-A-K--VV--GHCD--LDS-T----K--SCP-------------------
YP_001552293.1_  D---K--Y-NL--D-------------Q-WSILKKLL--------T-KLSAK-------Y-I-G-A-K--II--GHNE--I-S-N----K--SCPGFD---VQEWLQK-E----
CUR50697.1__22-  D---K--G-NP--Q------N-NFTPEQ-FASLK-LL----------LLAQKR----TY-P-N-A--K-IV--GHHD--LFA----G-K--ACP-------------------
ADX87661.1__16-  E---E--G-KA--D------A-NFTLEQ-YVSLKTLL--------D-TLKAS--------YP-E-A--E-IK--GHRD--V-N-N--G-K--ECP-------------------
AEI71112.1__23-  ----D-----F--A------R-NFTSSQ-DITLRQLIQG------I-SMRTR---------------I--RRISGHNQ--Y-A-A----K--ACPG------------------
AGC35529.1__26-  A---N--M-KA--Q------N-NFTSAQ-FATLAIKL--------R-ELKT--K-----Y-P-G-V--T-IQ--GHRD--F-AGV--K-K--DCP-------------------
YP_009216701.1_  IE-----DMPQ--V------Q-K----K-ALIELGIY--------LKNKYNI-------N-H-NNI-----M--GHRD--V-N----S-T--TCPGRL----------------
BAQ94242.1__26-  D--WK-QG-E---D--------NFTGEQ-WESLYKTL--------G-ELVAK-------Y-P-D-A--Q-IV--GHYMLDE-A------K--TCP-------------------
YP_009191815.1_  PF-----SAGP--E------A-HYTKDQ-LVSLVAVI--------K-ELLGK-------Y-P-G-A--E-VL--GHRN--A-D----PGK--QCP-------------------
```

```
YP_003347926.1_   E---E--G-KA--D------A-NFTLEQ-YVSLKTLL--------D-TLKAS--------YP-E-A--E-IK--GHRD--V-N-N--G-K--ECP------------------
YP_007005440.1_   A---S--G-KP--Q------A-NFTPQQ-MQSLRDVL--------A-GLKV--E-----Y-P-D-A--T-IH--GHRE--F-A----A-K--ACP------------------
YP_249586.1__27   E---E--G-KA--D------A-NFTLEQ-YVSLKTLL--------D-TLKAS--------YP-E-A--E-IK--GHRD--V-N-N--G-K--ECP------------------
ADX87518.1__16-   E---E--G-KA--D------A-NFTLEQ-YVSLKTLL--------D-TLKAS--------YP-E-S--E-IK--GHRD--V-N-N--G-K--ECP------------------
YP_004306332.1_   A---K--G-NP--E------D-NFTDHQ-KAALQTLL--------W-RMVS-GKDFDGAYQD-L-P--V-V---GHRD--L-D----PGK--ACP------------------
YP_009217953.1_   IE-----DMPQ--A------Q-K----K-ALIELGIY--------LKNKYNI-------N-H-NNI-----I--GHRD--V-N----P-T--SCPG-----------------
BAR16484.1__26-   -------G-----D--------NFTEDQ-MYTLHNVI--------Q-TIKDM-------H-PEI--EV--I---GHSD--V-E-P----KKPHCPG-----------------
YP_006488655.1_   I------------E--------KVTDAQ-YKALLWLIQ----------DIRN-R-------RGN-M--P-VY--GHKEL-N------S-T--DCPGNL---------------
BAQ86927.1__26-   -------G-----D--------NFTEDQ-MYTLHNVI--------Q-TIKDM-------H-PEI--EV--I---GHSD--V-E-P----KKPHCPG-----------------
YP_004286199.1_   D---S--G-KP--E------D-NFTDQQ-KAALSGLL-WDMTQSGV-TYGD--T-----YKE-L-P--V-V---GHRD--LDS----G-K--ACP------------------
BAQ86653.1__26-   -------G-----D--------NFTEDQ-MYTLHNVI--------Q-TIKDM-------H-PEI--EV--I---GHSD--V-E-P----KKPHCPG-----------------
ANS03019.1__21-   D---MKKA-K---D------T-R-TQAQ-KDSLIKLIH---------ELIYK-------Y-NKD-M-T--IH--GHNE--Y-A-N----K--ACP------------------
BAR33619.1__5-1   D---------------------LTETG-KEAILTVVE---------AMRNE-------Y-G-D-H-LW-VK--CHRD--F-G-T----T--YCPG-----------------

cons
```

Supplementary Fig. S7 Multiple sequence alignment of phage endolysins. The alignment shows the N- and C-terminal extensions in the thermophilic phage endolysins and demonstrates the uniqueness of these motifs for Ts2631 (AIM47292.1) and Ph2119 (AHF20915.1) endolysins The alignment was prepared using the T-coffee program available from http://www.tcoffee.org. The multiple sequence alignment is colored according to the transitive consistency score (TCS) scheme[5]. Dark pink blocks represent perfect transitive consistency between the T-Coffee library and the input alignment.

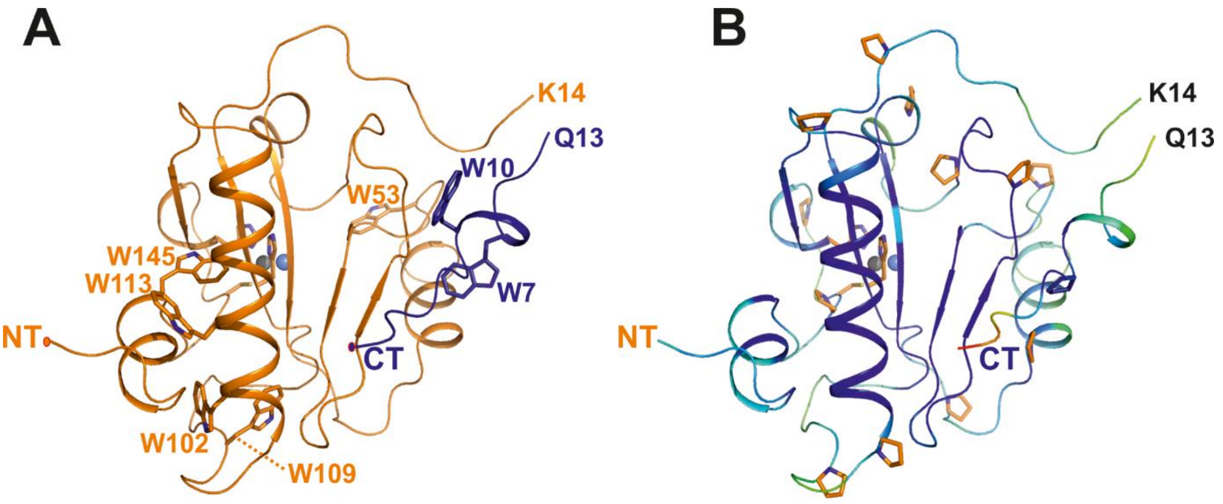

Supplementary Fig. S8 Distribution of overrepresented residues in Ts2631 endolysin. (A) The monomeric structure contains an unusually high number of tryptophans (seven), which are displayed in stick representation and distributed all over the structure, forming hydrophobic cores. (B) The structure (the color code from blue to red resembles the B-factors of Cα atoms) is shown with 13 prolines marked in stick representation. The prolines are distributed throughout the sequence and locally lower the B-factors in various structural elements.

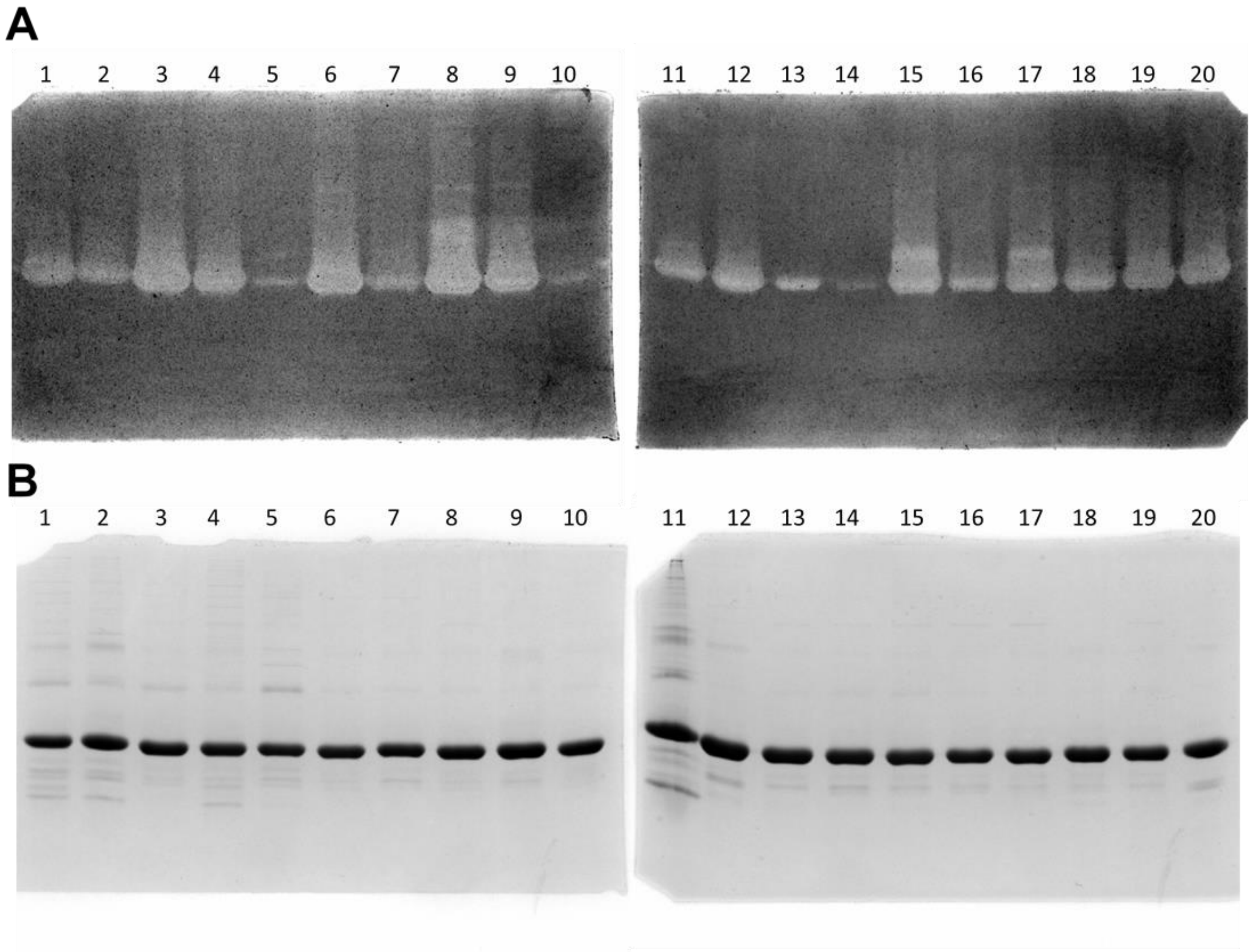

Supplementary Fig. S9 Lytic activity of Ts2631 endolysin variants at 37 °C. (A) Zymogram analysis of purified Ts2631 endolysin variants. (B) SDS-PAGE of purified variants (0.5 μg) served as a loading control for a zymogram assay. Numbers 1-10 correspond to H31A, T32A, A33G, P54A, Y60A, R64A, D65A, R67A, Y69A, and K70A (first gel in each panel), and numbers 11-20 correspond to L72A, I79A, C80A, N85A, G95A, D96A, N133A, V135A, E138A and native Ts2631 endolysin (second gel in each panel).

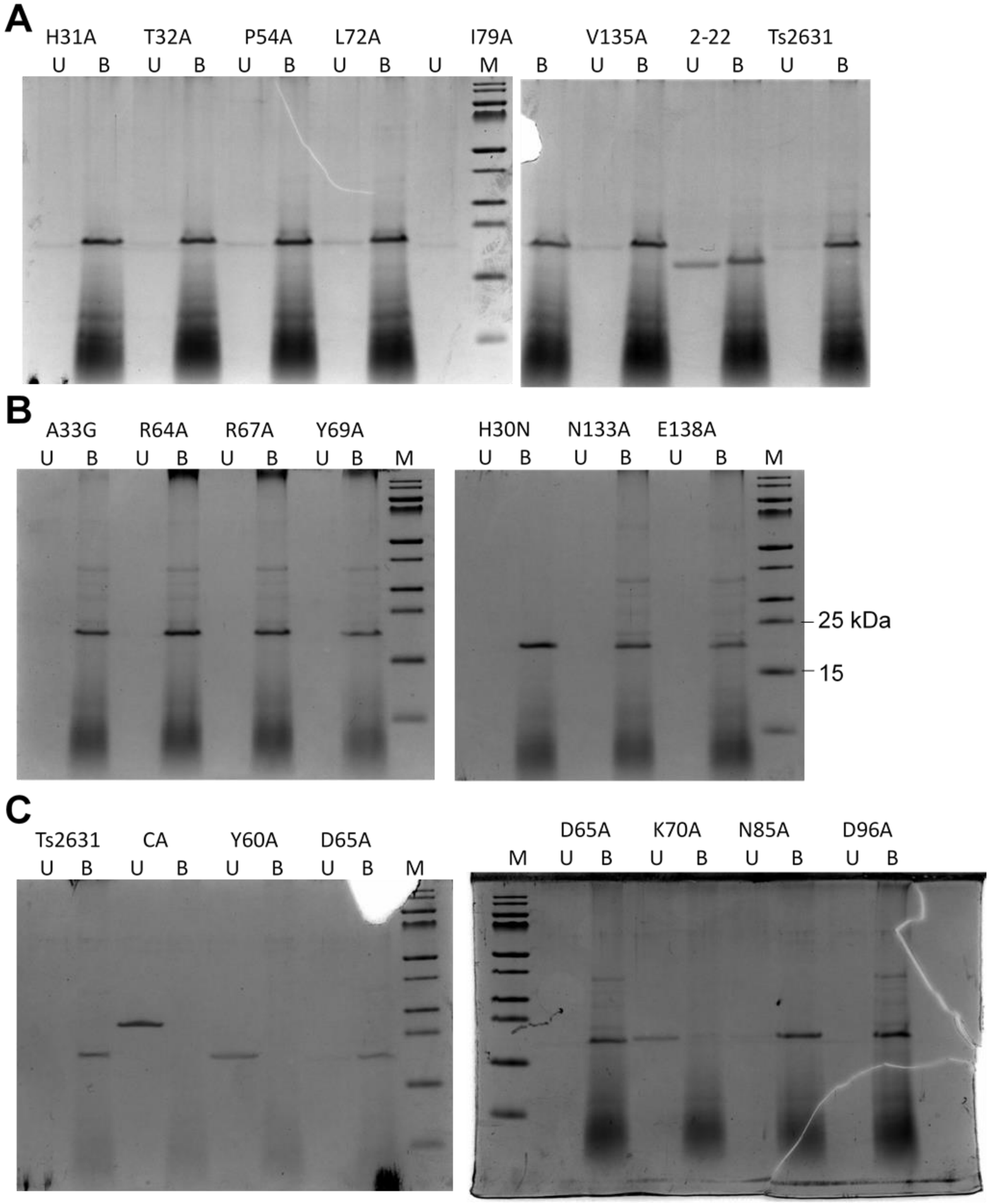

Supplementary Fig. S10 Evaluation of peptidoglycan binding activity of the Ts2631 endolysin variants (full-length selected gels). Ts2631 endolysin variants were mixed with purified *T. thermophilus* HB8 peptidoglycan, and the proteins remaining in the supernatant (*U*, unbound) or bound to peptidoglycan (*B*) were separated by centrifugation at 12,600 × g

for 5 min at 4 °C. Proteins were analyzed by SDS-PAGE. (A) Analysis of H31A, T32A, P54A, L72A, I79A, V135A, Ts2631Δ2-22 and a positive control - wild-type Ts2631 endolysin. (B) Analysis of A33G, R64A, R67A, Y69A, H30N, N133A and E138A. (C) Analysis of a positive control (wild-type Ts2631 endolysin), a negative control (carbonic anhydrase (CA)), Y60A, D65A (two repeats), K70A, N85A and D96A. M - PageRuler prestained protein ladder, 10 to 180 kDa (Thermo Fisher Scientific).

Supplementary Table S1 - PCR primers used for site-directed mutagenesis of the *ts2631* gene

Primer name:	Oligonucleotide sequences (5′→ 3′):	Change:
H31A_F	CGTTGTCCTGCACGCCACGGCGGGGCCG	CAC → GCC
H31A_R	CGGCCCCGCCGTGGCGTGCAGGACAACG	
T32A_F	TCCTGCACCACGCGGCGGGGCCG	ACG → GCG
T32A_R	CGGCCCCGCCGCGTGGTGCAGGA	
A33G_F	GCACCACACGGGGGGGCCGGAGA	GCG → GGG
A33G _R	TCTCCGGCCCCCCCGTGTGGTGC	
Y60A_F	TATCGGTTACCACGCTCTGGTCTACCGGG	TAT → GCT
Y60A_R	CCCGGTAGACCAGAGCGTGGTAACCGATA	
P54A_F	CCCGGGGGTGGGCCCATATCGGT	CCC → GCC
P54A_R	ACCGATATGGGCCCACCCCCGGG	
R64A_F	CACTATCTGGTCTACGCGGATGGGCGGGTGTA	CGG → GCG
R64A _R	TACACCCGCCCATCCGCGTAGACCAGATAGTG	
D65A_F	CTGGTCTACCGGGCTGGGCGGGTGTAC	GAT → GCT
D65A_R	GTACACCCGCCCAGCCCGGTAGACCAG	
R67A_F	GTCTACCGGGATGGGGCGGTGTACAAAACGCT	CGG → GCG
R67A_R	AGCGTTTTGTACACCGCCCCATCCCGGTAGAC	
Y69A_F	GGGATGGGCGGGTGGCCAAAACGCTCCCGA	TAC → GCC
Y69A_R	TCGGGAGCGTTTTGGCCACCCGCCCATCCC	
K70A_F	GGATGGGCGGGTGTACGCAACGCTCCCGAATAAC	AAA → GCA
K70A_R	GTTATTCGGGAGCGTTGCGTACACCCGCCCATCC	
L72A_F	GCGGGTGTACAAAACGGCCCCGAATAACGCCG	CTC → GCC
L72A_R	CGGCGTTATTCGGGGCCGTTTTGTACACCCGC	
I79A_F	CCCGAATAACGCCGTACCTGCTTGCGTTAGGGAGTTTAAC	ATT → GCT
I79A_R	GTTAAACTCCCTAACGCAAGCAGGTACGGCGTTATTCGGG	
C80A_F	CCGAATAACGCCGTACCTATTGCCGTTAGGGAGTTTA	TGC → GCC
C80A_R	TAAACTCCCTAACGGCAATAGGTACGGCGTTATTCGG	
N85A_F	CTATTTGCGTTAGGGAGTTTGCCCCTGTTTCCATTT	AAC → GCC
N85A_R	AAATGGAAACAGGGGCAAACTCCCTAACGCAAATAG	
G95A_F	GTGGCGGCGGTAGCGGATTTTTCAGCAG	GGG → GCG
G95A_R	CTGCTGAAAAATCCGCTACCGCCGCCAC	
D96A_F	GGCGGCGGTAGGGGCTTTTTCAGCAGGTG	GAT → GCT
D96A_R	CACCTGCTGAAAAAGCCCCTACCGCCGCC	

N133A_F	CGCTTTTTGTACTGCACAAAGCCCTTGTACCCACAG	AAC → GCC
N133A_R	CTGTGGGTACAAGGGCTTTGTGCAGTACAAAAAGCG	
V135A_F	ACTGCACAAAAACCTTGCACCCACAGAATGCCCTG	GTA → GCA
V135A_R	CAGGGCATTCTGTGGGTGCAAGGTTTTTGTGCAGT	
E138A_F	CTTGTACCCACAGCATGCCCTGGGCGG	GAA → GCA
E138A_R	CCGCCCAGGGCATGCTGTGGGTACAAG	
Δ2-22_F	CAACGTAATGGATAGGCATATGGCTGCCGCGC	del4-63
Δ2-22_R	GCGCGGCAGCCATATGCCTATCCATTACGTTG	
Δ2-29_F	CCGCCGTGTGGTGCATATGGCTGCCG	del4-84
Δ2-29_R	CGGCAGCCATATGCACCACACGGCGG	

Primers for site-directed mutagenesis of the *ts2631* gene were designed using the QuikChange Primer Design Program (http://www.genomics.agilent.com). Codons being changed are underlined.

References

1. Guan, R., Malchiodi, E. L., Wang, Q., Schuck, P. & Mariuzza, R. A. Crystal structure of the C-terminal peptidoglycan-binding domain of human peptidoglycan recognition protein Ialpha. *J Biol Chem* **279**, 31873-31882, doi:10.1074/jbc.M404920200 (2004).
2. Cho, S. *et al.* Structural insights into the bactericidal mechanism of human peptidoglycan recognition proteins. *Proc Natl Acad Sci U S A* **104**, 8761-8766, doi:10.1073/pnas.0701453104 (2007).
3. Marchler-Bauer, A. *et al.* CDD/SPARCLE: functional classification of proteins via subfamily domain architectures. *Nucleic Acids Res* **45**, D200-D203, doi:10.1093/nar/gkw1129 (2017).
4. Fischer, J. D., Mayer, C. E. & Söding, J. Prediction of protein functional residues from sequence by probability density estimation. *Bioinformatics* **24**, 613-620, doi:10.1093/bioinformatics/btm626 (2008).
5. Chang, J. M., Di Tommaso, P. & Notredame, C. TCS: a new multiple sequence alignment reliability measure to estimate alignment accuracy and improve

phylogenetic tree reconstruction. *Mol Biol Evol* **31**, 1625-1637, doi:10.1093/molbev/msu117 (2014).
